# Synthesis and Evaluation of ePSMA-DM1: A New Theranostic Small-Molecule Drug Conjugate (T-SMDC) for Prostate Cancer

**DOI:** 10.3390/ph16081072

**Published:** 2023-07-28

**Authors:** Erika Murce, Evelien Spaan, Savanne Beekman, Lilian van den Brink, Maryana Handula, Debra Stuurman, Corrina de Ridder, Simone U. Dalm, Yann Seimbille

**Affiliations:** 1 Department of Radiology and Nuclear Medicine, University Medical Center Rotterdam, Erasmus MC, 3015 GD Rotterdam, The Netherlands; e.murcesilva@erasmusmc.nl (E.M.); e.spaan@erasmusmc.nl (E.S.); s.beekman@erasmusmc.nl (S.B.); l.vandenbrink@erasmusmc.nl (L.v.d.B.); m.handula@erasmusmc.nl (M.H.); d.stuurman@erasmusmc.nl (D.S.); c.deridder@erasmusmc.nl (C.d.R.); s.dalm@erasmusmc.nl (S.U.D.); 2 Erasmus MC Cancer Institute, 3015 GD Rotterdam, The Netherlands; 3 TRIUMF, Life Sciences Division, Vancouver, BC V6T 2A3, Canada

**Keywords:** prostate cancer, PSMA, DM1, small-molecule drug conjugate, theranostics, targeted therapy, SPECT/CT imaging

## Abstract

Small-molecule drug conjugates (SMDCs) are compounds in which a therapeutic payload is conjugated to a targeting vector, for specific delivery to the tumor site. This promising approach can be translated to the treatment of prostate cancer by selecting a targeting vector which binds to the prostate-specific membrane antigen (PSMA). Moreover, the addition of a bifunctional chelator to the molecule allows for the use of both diagnostic and therapeutic radionuclides. In this way, the distribution of the SMDC in the body can be monitored, and combination therapy regimes can be implemented. We combined a glutamate-urea-lysine vector to the cytotoxic agent DM1 and a DOTA chelator via an optimized linker to obtain the theranostic SMDC (T-SMDC) ePSMA-DM1. ePSMA-DM1 retained a high binding affinity to PSMA and demonstrated PSMA-specific uptake in cells. Glutathione stability assays showed that the half-life of the T-SMDC in a reducing environment was 2 h, and full drug release was obtained after 6 h. Moreover, 100 nM of ePSMA-DM1 reduced the cell viability of the human PSMA-positive LS174T cells by >85% after 72 h of incubation, which was comparable to a 10-fold higher dose of free DM1. [^111^In]In-ePSMA-DM1 and [^177^Lu]Lu-ePSMA-DM1 were both obtained in high radiochemical yields and purities (>95%), with >90% stability in PBS and >80% stability in mouse serum for up to 24 h post incubation at 37 °C. SPECT/CT imaging studies allowed for a faint tumor visualization of [^111^In]In-ePSMA-DM1 at 1 h p.i., and the ex vivo biodistribution showed tumor uptake (2.39 ± 0.29% ID/g) at 1 h p.i., with the compound retained in the tumor for up to 24 h. Therefore, ePSMA-DM1 is a promising T-SMDC candidate for prostate cancer, and the data obtained so far warrant further investigations, such as therapeutic experiments, after further optimization.

## 1. Introduction

Metastatic, castration-resistant prostate cancer (mCRPC) is an advanced form of prostate cancer in which tumors are no longer responsive to androgen deprivation therapy. Current treatments are palliative in nature and aim to prolong survival while maintaining quality of life [1]. mCRPC patients are usually heavily treated with chemotherapeutic agents, known to present side effects due to their lack of selectivity and narrow safety profile [2]. Therefore, there is a critical need to develop effective treatments with reduced toxicity for this group of patients. 

Recently, increased attention has been given to the development of radioligand therapy (RLT) using radiopharmaceuticals targeting the prostate-specific membrane antigen (PSMA) [3], a target commonly overexpressed in prostate cancer and whose expression increases with disease progression [4]. These agents are designed to deliver cytotoxic radiation directly to prostate cancer cells via a therapeutic payload (a radionuclide emitting beta or alpha radiation) connected to a targeting vector which binds specifically to PSMA. Such a targeted approach reduces the toxicity to healthy tissues and therefore the side effects for patients [5]. By using the same targeting vector in combination with a diagnostic radionuclide before treatment, a theranostic approach can be applied [6]. 

While PSMA-mediated RLT has shown clinical success thus far, illustrated by the FDA approval of Pluvicto™ ([^177^Lu]Lu-PSMA-617), patients eventually develop resistance and disease recurrence [7]. Combination strategies are currently being investigated to improve the therapeutic efficacy of RLT, such as the use of radiosensitizers [8], androgen receptor targeting drugs [9,10] and immunotherapy [11]. The combination with chemotherapeutic agents is also an interesting approach, as it is based on the rationale of creating additional DNA damage in order to increase the efficacy of the treatment. Chemotherapy also improves tumor reoxygenation, leading to increased cell sensitivity towards radiation. This combination may also be particularly effective in heterogenous tumors, as non-PSMA expressing cells will also be targeted [12]. Several small-scale clinical studies combining RLT and chemotherapeutic agents have shown that this approach is efficient against tumors [13]. However, there is currently only one clinical study investigating the combination of RLT with chemotherapy for prostate cancer, UpFrontPSMA (NCT04343885), in which patients with metastatic hormone-naïve prostate cancer receive either a combination of docetaxel (six cycles, every 3 weeks) with [^177^Lu]Lu-PSMA-617 (two sequential doses, 6 weeks apart), or docetaxel alone. As such, new studies to elucidate the potential of the combination of these different therapeutic regimes are still needed.

Due to the high systemic toxicity of common chemotherapeutics, we focused our attention on targeted drug delivery. A successful approach so far has been the use of antibody–drug conjugates (ADCs), as illustrated by the market approval of several agents [14]. ADCs consist of a therapeutic payload attached to an antibody targeting a specific protein expressed in tumor cells. Due to the high specificity of the antibody, this approach reduces the toxicity of the cytotoxic agent to non-targeted organs. While there are currently no approved ADCs for the treatment of prostate cancer, several promising conjugates are being evaluated in clinical trials. Nonetheless, elevated toxicity remains a limiting factor during the clinical evaluation of these agents [15,16], and many fail to further advance clinically. In addition, the disadvantages of ADCs are their suboptimal distribution and kinetics in vivo, slow clearance from the body, reduced tumor penetration and the immunogenic response they elicit. 

Small-molecule drug conjugates (SMDCs) are emerging as an interesting alternative to ADCs [17]. These drugs employ small molecules instead of antibodies, allowing for faster clearance and better tumor penetration. The synthesis of these conjugates can also be controlled via the attachment of different components using site-specific chemical reactions, ensuing manufacturing conformity. The basic structure of these compounds includes a targeting ligand, a linker and the therapeutic payload. In order to efficiently and selectively deliver the payload, the targeting ligand must bind strongly to its target. The linker consists of a spacer and can contain a cleavable bond to release the therapeutic payload. The spacer allows for sufficient distance between the therapeutic payload and ligand and can modulate the properties of the conjugate (i.e., hydrophilicity) to improve its pharmacokinetics. If a cleavable bond is present, it releases the cytotoxic agent specifically at the tumor site, to prevent off-target toxicity. Currently, a few SMDCs targeting PSMA have been described in the literature, featuring therapeutic payloads such as MMAE [18,19,20], tubulysin [21,22], topoisomerase inhibitors [23] and taxanes [24,25]. Some of these agents also include a chelator for the complexation of a radionuclide to enable companion diagnostics [26]. This new class of drug is named theranostic small-molecule drug conjugates (T-SMDCs).

The success of a SMDC is greatly reliant on the choice of cytotoxic agent. Mertansine, also known as DM1, is a highly cytotoxic member of the maytansinoid family [27]. DM1 is a microtubule-disrupting agent; it binds to tubulin in order to block the microtubule assembly. This leads to a disruption of mitosis, and ultimately to cell death by inducing apoptosis [28]. A disadvantage of the standalone use of DM1 is its highly cytotoxic nature, leading to systemic toxicity, as well as severe adverse effects. This could be bypassed by coupling DM1 to a targeting vector. The conjugate acts as a pro-drug, by being selectively cleaved in the tumor microenvironment to release the drug specifically at the disease site. The presence of a sulfhydryl group in the drug allows for facile drug conjugation via a disulfide bridge to any thiol-containing targeting vector. DM1 has been successfully used as the payload of choice for several ADCs, including the market-approved trastuzumab emtansine for the treatment of breast cancer [29], and has also been evaluated in promising SMDCs [30,31,32].

**Figure 1:**
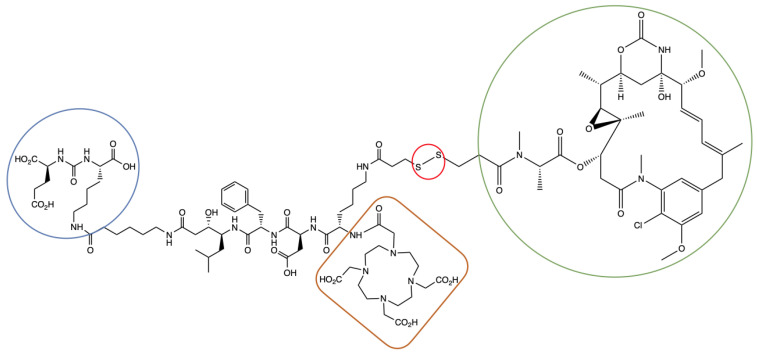
Chemical structure of ePSMA-DM1. The PSMA-targeting moiety EuK (delineated by the blue circle) is attached via an amino acid linker (Ahx-Sta-Phe-Asp-Lys) to both a DOTA chelator (delineated in orange) and the DM1 drug (delineated in green) via a disulfide bond linkage (delineated in red).

To our knowledge, the use of a single molecule containing both a therapeutic radionuclide and a chemotherapeutic payload has not yet been reported in the literature. Furthermore, the biodistribution and pharmacokinetics of the drug conjugate, used for both RLT and as an SMDC, could be easily studied by employing a diagnostic radionuclide. A synergistic effect may also be achieved by combining the two therapeutic regimens, enhancing the efficacy of the drug conjugate. With this in mind, we designed, synthesized and performed a preliminary biological evaluation of the PSMA-targeting T-SMDC, ePSMA-DM1, consisting of (i) a Glu-urea-Lys (EuK) moiety, known to bind strongly to PSMA; (ii) an optimized linker to improve the pharmacokinetic properties; (iii) a 2,2′,2″,2‴-(1,4,7,10-tetraazacyclododecane-1,4,7,10-tetrayl)tetraacetic acid (DOTA, also known as Tetraxetan) chelator, which can complex a wide variety of radionuclides, such as In-111 (for imaging) and Lu-177 (for therapy); and (iv) the cytotoxic agent DM1 conjugated via a cleavable disulfide linker (Figure 1). The conjugate was evaluated for its binding to PSMA and its cytotoxicity in PSMA-expressing LS174T cells. Radiolabeling with the diagnostic radionuclide In-111 and the therapeutic radionuclide Lu-177 was performed, and their stability in PBS and mouse serum was evaluated. Cell uptake and internalization experiments were performed using the PSMA-positive human cancer cell line LS174T with the indium-radiolabeled conjugate. SPECT-CT imaging and ex vivo biodistribution studies were performed in BALB/c nu/nu LS174T xenografted mice with an indium-labeled probe to elucidate the compound’s biodistribution profile. 

## 2. Results

**Scheme 1:**
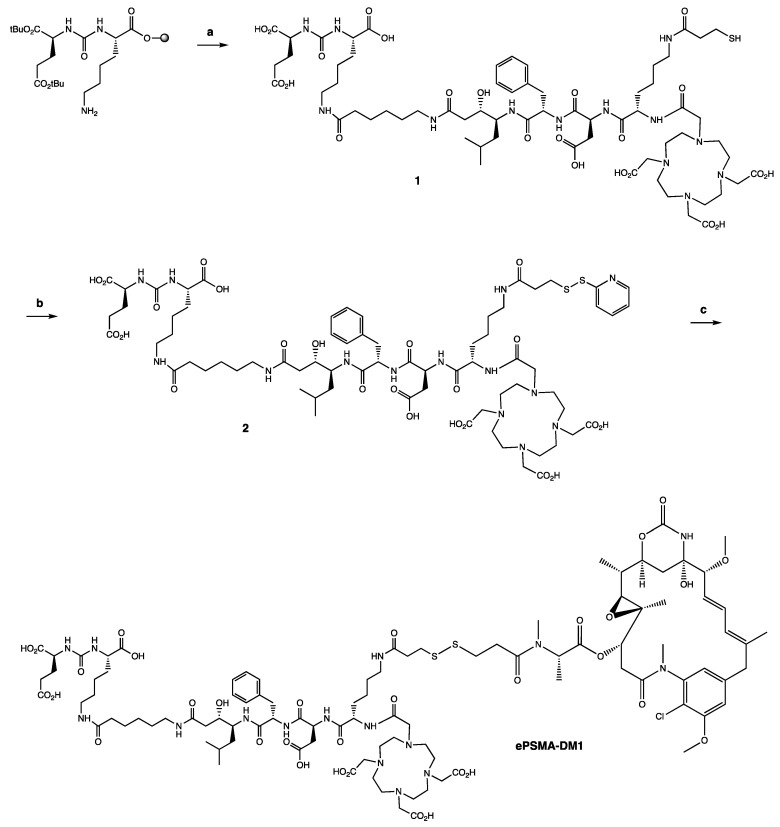
Synthesis of ePSMA-DM1. (**a**) (i) Fmoc-6-Ahx-OH, HBTU, Oxyma Pure, DIPEA, DMF, 45 min, rt; (ii) 20% piperidine in DMF, 3 × 5 min, rt; (iii) Elongation with Fmoc-*3S*,*4S*-Sta-OH, Fmoc-L-Phe-OH, Fmoc-Asp(*t*Bu)-OH, ivDde-Lys(Fmoc)-OH, and 3-(tritylthio)propionic; (iv) 5% hydrazine in DMF, 2 × 15 min, rt; (v) DOTA(*t*Bu)_3_ ester, PyBOP, DIPEA, DMF, o.n., rt; and (vi) TFA/TIS/H_2_O, 2 h, rt, 22%. (**b**) (i) 2,2-dipyridyldisulfide, acetic acid, DMF, 15 min, rt; (ii) Aqueous NaOAc (0.2 M), dropwise for 5 min, rt; 30 min, rt, 73%. (**c**) DM1, Et_3_N, DMF, 15 min, rt, 66%.

The intermediate **1** consisted of the PSMA-targeting moiety EuK and a short amino acid linker containing a N-terminal lysine residue. The lysine was used as an anchor point for the conjugation of the DOTA chelator at the α-NH_2_ position and the incorporation of a mercaptopropionic acid linker at the ε-NH_2_ position. The sulfhydryl group was subsequently exploited to attach the cytotoxic drug DM1. The multi-step SPPS provided **1** with a yield of 22% after cleavage from solid support and HPLC purification (Scheme 1). The conjugation of DM1 was performed by a two-step approach via a thiol-disulfide exchange using 2,2-dipyridyldisulfide. ePSMA-DM1 was obtained with a good yield of 48% over the last two steps and a high chemical purity (>95%) after HPLC purification. All compounds were characterized by LC/MS (Figures S1.1–S1.3).

To assess the stability of the PSMA-targeted T-SMDC, we radiolabeled ePSMA-DM1 with In-111 and Lu-177. Both [^111^In]In-ePSMA-DM1 and [^177^Lu]Lu-ePSMA-DM1 were obtained in high radiochemical yields (RCYs) and radiochemical purities (RCPs) at a molar activity of 50 MBq/nmol, when labeling was performed at 90 °C for 5 min (Table 1). [^111^In]In-ePSMA-DM1 was stable in PBS (90.9%) and mouse serum (82.9%) for up to 24 h post radiolabeling (Figures S2 and S3). [^177^Lu]Lu-ePSMA-DM1 was also found to be stable in these media (81.4% and 91.2% were intact in PBS and mouse serum, respectively) 24 h post radiolabeling (Figures S5 and S6). [^111^In]In-ePSMA-DM1 was highly hydrophilic, as demonstrated by the logD value (−2.47 ± 0.11) obtained by the shake-flask method. 

**Figure 2:**
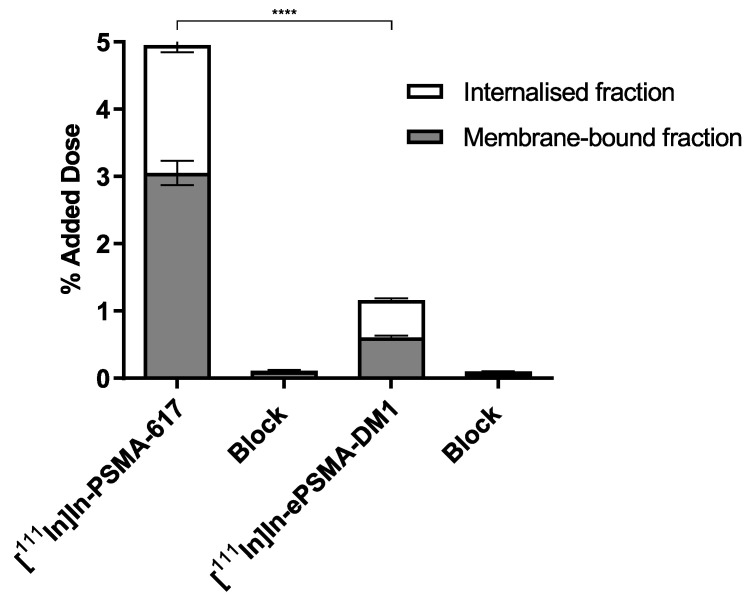
Uptake and internalization of [^111^In]In-PSMA-617 and [^111^In]In-ePSMA-DM1 in PSMA-expressing LS174T cells. Values are expressed as % of the added dose per 1.000.000 cells (% AD). Compounds were blocked with a 50-fold excess of PSMA-617. Statistically significant differences (*p*-value) are indicated by asterisks (four (****) asterisks indicate *p* < 0.0001).

The binding of ePSMA-DM1 to PSMA was evaluated with an NAALADase enzymatic assay using PSMA-617 as an internal reference. ePSMA-DM1 exhibited a binding affinity of 45.3 ± 1.84 nM, compared to 2.19 ± 0.78 nM for PSMA-617 (Figure S7). It demonstrated that the compound retained a nanomolar binding affinity, despite the 20-fold loss in affinity likely due to the conjugation of the bulky DM1 drug. Next, we evaluated the uptake and internalization of [^111^In]In-ePSMA-DM1 in PSMA-expressing LS174T cells (Figure 2). [^111^In]In-ePSMA-DM1 showed PSMA-specific uptake with 48% corresponding to the internalized fraction (0.61 ± 0.07% added dose (AD) membrane-bound and 0.55 ± 0.07% AD internalized). The cell uptake of [^111^In]In-ePSMA-DM1 was significantly reduced when blocked with an excess of unlabeled PSMA-617 (0.06 ± 0.02% AD membrane-bound and 0.04 ± 0.01% AD internalized). The total uptake of [^111^In]In-ePSMA-DM1 was 4.3-fold lower than the uptake of the reference [^111^In]In-PSMA-617 (1.16 ± 0.14% AD vs. 4.95 ± 0.83% AD, respectively). 

**Figure 3:**
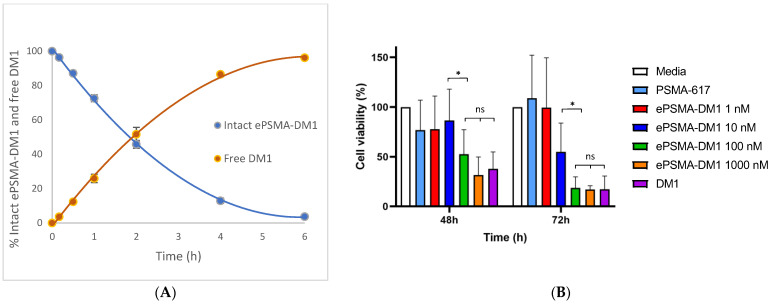
(**A**) Results of the glutathione stability assay. The curve in blue represents the percentage of intact ePSMA-DM1 over time in reductive conditions, while the curve in orange represents the free DM1 generated. (**B**) Percentage of PSMA-positive LS174T cell viability after incubation with ePSMA-DM1 or DM1 for 48 and 72 h. Media and PSMA-617 (100 nM) were used as negative controls. A concentration of 1000 nM of DM1 was used as a positive control, and 4 different doses of ePSMA-DM1 (1, 10, 100 and 1000 nM) were evaluated. Statistically significant differences (*p*-value) are indicated by asterisks (one (*) asterisks indicate *p* < 0.05). When no significant difference was observed, data is indicated by *ns*.

The ability of ePSMA-DM1 to release the drug in a reducing environment was evaluated in vitro via a glutathione (GSH) stability assay (Figure 3A). After 2 h, ~50% of the drug was released when ePSMA-DM1 was incubated in a medium containing 5 mM GSH, while full release occurred at 6 h. Then, we studied the efficacy of ePSMA-DM1 at killing LS174T cells by a resazurin-based cytotoxicity assay (Figure 3B). Four different concentrations of ePSMA-DM1 (1, 10, 100 and 1000 nM) were used to determine the minimum dose at which a cytotoxic effect could be observed. The free drug DM1 (1000 nM) was used as a positive control, while both media and PSMA-617 (100 nM) were used as negative controls. At 48 h post treatment, a cell viability of ~50% was observed for the two highest doses of ePSMA-DM1 and the free DM1. In fact, no statistical difference was found at 48 h post treatment between the cytotoxic effect of 100 nM of ePSMA-DM1 and 1000 nM of DM1 (*p* = 0.16), indicating that a 10 × lower dose of ePSMA-DM1 was as potent as the free drug. However, a significant difference was found when comparing the effect of the 10 nM dose of ePSMA-DM1 and 1000 nM of DM1 (*p* = 0.02). It was further confirmed at 72 h, since the total cell viability decreased by more than 85% for the 100 and 1000 nM doses of ePSMA-DM1, as well as 1000 nM of DM1. At 72 h, 50% cell death was observed for the 10 nM dose of ePSMA-DM1, while this dose had initially no significant effect on cell viability (*p* = 0.21) when compared to the media and PSMA-617. However, the dose of 1 nM of ePSMA-DM1 was not sufficient to elicit cell death, remaining statistically identical to the negative controls at both time points. 

**Figure 4:**
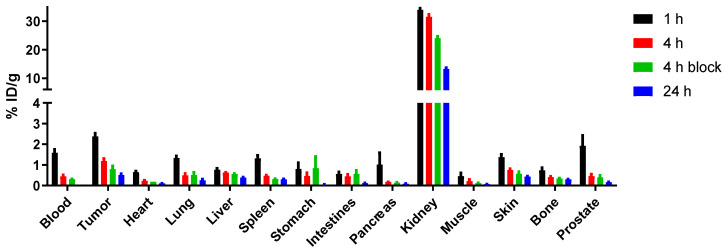
Ex vivo biodistribution of [^111^In]In-ePSMA-DM1 in BalB/C nu/nu mice xenografted with PSMA-positive LS174T tumor cells in the right flank. Mice were injected with ~5 MBq/1 nmol and euthanized at 1, 4 and 24 h p.i. A block group was also euthanized at 4 h p.i. Uptake is expressed as the percentage of injected dose per gram of tissue collected.

Mice were injected with either 5.19 ± 0.34 MBq (1.04 nmol) or 19.40 ± 1.49 MBq (0.97 nmol) of [^111^In]In-ePSMA-DM1 for ex vivo biodistribution and in vivo SPECT/CT imaging, respectively. The tumor uptake of [^111^In]In-ePSMA-DM1 measured in the ex vivo biodistribution was 2.39 ± 0.29% ID/g, 1.19 ± 0.22% ID/g and 0.50 ± 0.14% ID/g at 1, 4 and 24 h p.i., respectively. To verify PSMA specificity, a 50-fold excess of unlabeled PSMA-617 was co-injected with the activity. At 4 h p.i., a high uptake of 0.80 ± 0.31% ID/g was still observed in the blocking group (vs. 1.19 ± 0.22% ID/g non-blocked at 4 h p.i., not significantly different, *p* = 0.15), suggesting that the uptake of ePSMA-DM1 was not exclusively PSMA-mediated. Elevated kidney uptake, which is characteristic of PSMA-targeting radiopharmaceuticals, was also observed (33.97 ± 2.54% ID/g at 1 h p.i., decreasing significantly to 13.23 ± 0.95% ID/g at 24 h p.i., *p* = 0.001). The renal uptake was also partially blocked at 4 h (31.55 ± 1.78% ID/g unblocked group vs. 24.02 ± 1.46% ID/g blocked group, *p* = 0.002). At 1 h p.i., the compound was still circulating in the blood (1.43 ± 0.04% ID/g) but was rapidly cleared (0.37 ± 0.01% ID/g and 0.04 ± 0.00% ID/g at 4 and 24 h p.i., respectively), while it was slowly cleared from the off-target organs. The tumor-to-kidney ratios were the highest at 1 h p.i., then decreased to half at 4 h and remained constant up to 24 h p.i. The tumor-to-muscle ratios were constant throughout the experiment. The data are summarized in Figure 4, and the values for the complete ex vivo biodistribution studies can be found in Table S1.

**Figure 5:**
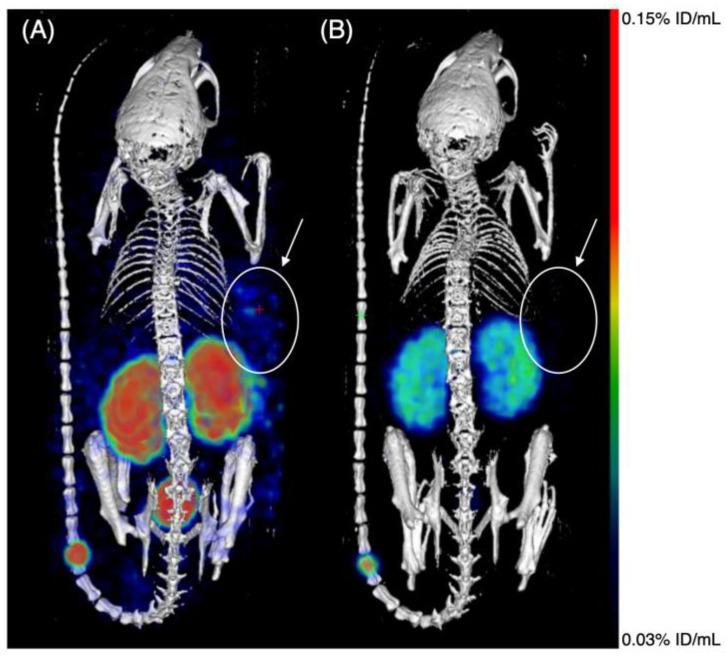
Representative MIP images of SPECT/CT scans after injection of [^111^In]In-ePSMA-DM1 at (**A**) 1 h p.i. and (**B**) 24 h p.i. in LS174T xenograft-bearing mice (right flank; n = 4). The white arrow and circle delineate the tumor. Color scale represents % ID/mL (min. = 0.03% ID/mL, max. = 0.15% ID/mL).

SPECT/CT imaging showed a high uptake in the bladder and the kidneys at 1 h p.i., with a faint tumor uptake (Figure 5). Background signal was seen in the abdominal region, which could be explained by the blood circulation of the tracer, as confirmed by the ex vivo biodistribution. At 24 h p.i., the tumor could not be visualized, and only moderate signal in the kidneys was observed.

## 3. Discussion

In this study, we aimed to develop a PSMA-targeting theranostic small-molecule drug conjugate (T-SMDC) for the imaging of and combined therapy for prostate cancer. SMDCs are emerging as a form of targeted chemotherapy, with several promising SMDCs targeting PSMA being reported in the literature [18,19,20,21,22,23,24,25]. However, SMDCs must be carefully designed in order to maximize their therapeutic efficacy. We designed the compound ePSMA-DM1 based on the targeting moiety EuK, known to bind strongly to PSMA [33,34]. Indeed, the T-SMDC must have a high binding affinity to the target, so that a lower concentration of the drug is required to elicit its therapeutic effect [26]. The rational design of the spacer (Ahx-Sta-Phe-Asp) was based on the binding pocket of the target. Moreover, a hydrophilic linker was desired to modulate the pharmacokinetics of the compound, as the therapeutic payload is highly lipophilic. An additional lysine residue was added to the linker Ahx-Sta-Phe-Asp for the conjugation of the DOTA chelator at the terminal α-NH_2_, while a short thiolated linker was introduced at the ε-NH_2_ position. The sulfhydryl group was used to conjugate DM1 via a disulfide bridge. Solid-phase peptide synthesis (SPPS) allowed for a facile synthesis of the peptide up to the attachment of the DOTA chelator. The final compound, ePSMA-DM1, was obtained via two liquid-phase steps with a good yield. 

The cytotoxicity is dependent on the choice of the therapeutic payload and the ability of the conjugate to adequately transport and release it at its intended site of action. Cleavable and non-cleavable linkers may be used in the design of SMDCs. However, conjugates containing cleavable linkers have been shown to be more effective than the corresponding analogs containing non-cleavable linkers [20,35]. The linker must be carefully selected to remain intact during circulation, and to release the drug at the site of disease. Premature drug release often leads to systemic toxicity, as well as decreased drug efficacy. An example is neutropenia, a commonly reported toxicity in ADCs containing the valine-citruline protease cleavable linker, due to the premature release of the drug and action of the cytotoxic payload in the bone marrow [36]. Another frequently employed cleavable linker is the disulfide bond, which is cleaved under reductive conditions. Tumor cells contain a higher concentration (100–1000× fold higher) of glutathione (GSH) compared to the extracellular concentration. GSH is a potent reductive agent, reacting readily with disulfide bonds [37,38]. A GSH stability assay was used to mimic the intracellular release of the cytotoxic payload. We determined that DM1 was fully released from ePSMA-DM1 within 6 h. Compared to other DM1-containing SMDCs, the release of the payload takes longer for our compound, even though the disulfide linker is not sterically hindered [30].

Using an NAALADase enzymatic assay to evaluate binding to PSMA, we determined that ePSMA-DM1 had an IC_50_ value approximately 20-fold worse than the IC_50_ value of PSMA-617 (45.3 ± 1.84 nM vs. 2.19 ± 0.78 nM). While both compounds are based on the EuK binding moiety, structural modifications in the linker region and the addition of the drug affected the binding due to steric hindrance or unfavorable interactions inside the binding pocket of the protein [39,40]. The radiochemical evaluation of ePSMA-DM1 with In-111 and Lu-177 also showed that the compounds were stable in formulation buffer (PBS) and mouse serum, confirming that the PSMA-binding unit attached to DM1 via a disulfide bridge was stable towards radiolysis and proteolytic degradation.

The cell uptake of [^111^In]In-ePSMA-DM1 was 4.3-fold lower than the cell uptake of the reference [^111^In]In-PSMA-617, which was expected due to the lower binding affinity. This is attributed to the attachment of the bulky cytotoxic payload, as previously reported for other SMDCs [21,23,35]. However, the ratio of internalization was slightly increased for [^111^In]In-ePSMA-DM1 (48% compared to 38% for [^111^In]In-PSMA-617), which is desired as the cytotoxic payload has to be internalized to exert its effect on tubulin [29,30]. Blocking significantly reduced the cell uptake of [^111^In]In-ePSMA-DM1, proving that the uptake was PSMA specific. 

Cytotoxicity studies were then performed in LS174T cells. We observed high variability in our assay, particularly for the lower doses of ePSMA-DM1 and PSMA-617. It could be due to all the washes included in our experimental protocol or the use of the kit, which led to variable fluorescence values [41]. The rapid cell growth could also have an effect on the variability observed (a doubling time of 30–40 h). However, the number of cells used in the assay was optimized to avoid confluency. Despite the variability, we observed a decrease in viability for the dose of 10 nM of ePSMA-DM1. This was not observed with the lowest dose of 1 nM or the negative control PSMA-617 (100 nM). However, at both timepoints, the doses of 100 and 1000 nM of ePSMA-DM1 and the dose of 1000 nM DM1 had similar cytotoxicity (*p* = 0.36; ANOVA). These results showed that ePSMA-DM1 was 10-fold more potent than DM1, confirming that conjugation to the PSMA binding moiety increased the cytotoxicity of the payload. It was particularly encouraging since a certain degree of cytotoxicity is usually preserved when the drug is conjugated to a targeting vector, but the free drug is often more potent than the SMDCs [19,30,35]. 

The results of the ex vivo biodistribution of [^111^In]In-ePSMA-DM1 showed that the tumor uptake was the highest at 1 h p.i. (2.39 ± 0.29% ID/g) with around half of the activity still present at 4 h p.i (1.19 ± 0.22% ID/g). Therefore, we estimated that the half-life of the T-SMDC in the tumor was 2.1 h. Consequently, the majority of the drug will be released while the T-SMDC is bound to the tumor cells. In fact, 50% of the drug was released after 2 h of incubation of ePSMA-DM1 in the presence of a physiologic concentration of GSH. Thus, the tumor retention and cleavage of the conjugate were in good accordance. A faster release could increase the risk of undesired premature drug release in circulation. This concern is justified, as [^111^In]In-ePSMA-DM1 was still present in circulation at 1 h p.i. (1.43 ± 0.04% ID/g). The stability of the linker can be easily modulated by incorporating substituents adjacent to the disulfide bridge. This has been shown to increase the stability of this bond, supposedly due to a steric hindrance effect, without hindering the cytotoxicity of the conjugate. Alternatively, increasing the size of the aliphatic carbon chain adjacent to the disulfide bridge may contribute to faster release kinetics [42]. These strategies could be used to further modulate the drug release of ePSMA-DM1. 

At 1 h p.i., the compound was still circulating in the blood (1.43 ± 0.04% ID/g) but was rapidly cleared (0.37 ± 0.01% ID/g and 0.04 ± 0.00% ID/g at 4 and 24 h p.i., respectively). The half-life of [^111^In]In-ePSMA-DM1 in the blood was estimated to be 1.5 h. We did not perform exsanguination during our biodistribution; therefore, residual blood could still be present in the different tissues during collection. This could explain the uptake observed in organs such as the lung, spleen, stomach, pancreas, skin and prostate at the 1 h timepoint. However, clearance in these organs was also observed. Furthermore, a reduction in the disulfide bond is required for ePSMA-DM1 to exert its cytotoxic effect. Therefore, no additional toxicity to these non-targeted organs is anticipated due to the absence of the required reductive environments. Nonetheless, further metabolite studies are necessary to confirm that indeed no reduction takes place.

Partial uptake was also observed in the blocked group. It might be explained by the existence of a different mechanism of cell uptake, which acts concomitantly with the PSMA-mediated uptake. However, in our cell uptake and internalization studies, we barely observed any uptake in the blocked group, suggesting that the compound’s uptake was somehow PSMA specific. Nevertheless, a limitation of such an experiment is the use of a single PSMA-expressing cell line, which does not allow us to predict the systemic effect of the tracer and its uptake in different organs [43]. Moreover, in our previous study, we reported the ex vivo biodistribution of [^111^In]In-**22**, consisting of the same targeting moiety and linker, EuK(Ahx-Sta-Phe-Asp), to which DOTA-GA was conjugated. [^111^In]In-**22** showed low off-target accumulation, and the tumor uptake could be blocked by an excess of PSMA-617 [44]. However, [^111^In]In-ePSMA-DM1 differs from [^111^In]In-**22** by the addition of a lysine in the spacer and the conjugation of DM1 onto the side chain of the lysine residue. Notably, **22** showed a considerably better binding affinity to the PSMA (IC_50_ = 1.66 ± 0.63 nM) than ePSMA-DM1, which raises the question of whether the lower binding affinity could justify the decreased specificity of [^111^In]In-ePSMA-DM1, as previously described [45].

The off-target accumulation of SMDCs has already been reported in the literature. The first PSMA-targeting theranostic SMDC was designed by Kumar et al. [31]. ^68^Ga-labeled NO3A-DM1-Lys-Urea-Glu consisted of the EuK PSMA-targeting moiety, DM1 as a cytotoxic agent and an NO3A chelator for PET imaging. High uptake was observed in PSMA-positive PC3-PIP tumors (4.30 ± 0.20% ID/g) at 1 h p.i. with clear tumor visualization, while the uptake in PSMA-negative PC3-Flu tumors was lower (1.12 ± 0.42% ID/g), but still significant. Similarly to what was observed for [^111^In]In-ePSMA-DM1, ^68^Ga-labeled NO3A-DM1-Lys-Urea-Glu also showed some uptake in non-targeted organs (e.g., liver, 0.42 ± 0.10% ID/g at 1 h p.i.). While no further results have been published regarding this compound, the same group also reported a tri-modal platform, in which the EuK targeting moiety, a prosthetic group for ^18/19^F-labeling and a cytotoxic agent (TLR7 agonist, connected via a legumain cleavable linker) were connected via a triazine group [46]. Off-target accumulation was observed, with elevated heart, lung, kidney and liver uptake, presumably due to the lipophilic nature of the construct. While the uptake of the conjugate in PSMA+ PC3-PIP tumors (1.9 ± 0.4% ID/g) was higher than in PSMA- PC3-Flu tumors (0.8 ± 0.3% ID/g), these results still indicated the conjugate’s considerable non-specificity. 

Lv et al. also reported a DUPA-based PSMA-targeting SMDC conjugated to paclitaxel, either with or without a cleavable bond (PTX-SS-DUPA and PTX-DUPA, respectively) [35]. PTX-DUPA showed no significant antitumor effect, illustrating the importance of the cleavable bond for the drug to elicit its effect. PTX-SS-DUPA showed tumor and off-target accumulation. However, the presence of the PSMA-targeting moiety led to an improved tumor-to-lung ratio compared to the free paclitaxel, as determined by the high uptake in this organ (uptake of PTX: 2.81 ± 0.62 μg/g tissue in tumor; 6.35 ± 0.46 μg/g tissue in lung; uptake of PTX-SS-DUPA: 0.65 ± 0.13 μg/g tissue in tumor; 0.28 ± 0.14 μg/g tissue in lung). Surprisingly, the tumor volume of the PTX-SS-DUPA-treated mice decreased significantly and was statistically significant to the tumor volume decrease of mice treated with free paclitaxel (using the same dose of both drugs), despite the difference in uptake observed in the tissue distribution studies. No significant toxicities were observed in the mice during the treatment course, nor were any histological changes observed after H&E staining of organ sections. In other studies of PSMA-targeting SMDCs that included therapeutic efficacy experiments, but not distribution studies, systemic toxicity was not observed either [19,20]. These results demonstrated that, while the SMDC strategy globally leads to a reduction in the toxicity and improved therapeutic efficacy, off-target uptake is always observed when using an SMDC. Despite observing promising results from the in vitro cytotoxicity of ePSMA-DM1, we cannot disregard the off-target uptake observed in vivo with [^111^In]In-ePSMA-DM1, which would hamper therapeutic studies with Lu-177. Nevertheless, as the cleavage of the disulfide bond is required for the drug to elicit its action, the off-target uptake might not thwart the use of the non-radioactive analog for targeted chemotherapy. However, to fully benefit from the potential of the T-SMDC, further optimization of the design of ePSMA-DM1 is required to reduce the off-target accumulation of the radioconjugate before performing therapeutic studies. 

T-SMDCs are promising emerging therapies, as they allow targeted treatment in combination with imaging prior to and during therapy to predict and monitor tumor response. Nevertheless, the results obtained here suggest that the mechanisms governing the uptake of SMDCs are still not entirely elucidated and that the use of targeting vectors to specifically convey drugs to tumors is not as straightforward as expected. While these compounds show promise, minimal off-target toxicity should be observed for clinical translation. This is even more important when considering therapeutic combinations, as other treatments will also bring in their own toxicity profiles, which are combined with the side effects of the SMDC therapy. Before further therapeutic studies can be carried out, a better understanding of the biological effect of our T-SMDC should be investigated. This is particularly important when considering combination regimes of ePSMA-DM1 and [^177^Lu]Lu-ePSMA-DM1, as considerable toxicity to the kidneys and salivary glands has been observed in patients undergoing [^177^Lu]Lu-PSMA therapy. The additional treatment with the cytotoxic drug may give rise to unpredictable side effects.

## 4. Materials and Methods

### 4.1. General Methods

#### 4.1.1. Chemistry

Solvents and chemicals were obtained from commercial suppliers in reagent grade or better and were used without further purification unless specified. Manual peptide synthesis vessels (Chemglass; Vineland, NJ, USA) were used to perform Fmoc-based solid-phase peptide synthesis (SPPS). Kaiser and/or TNBS tests were used to monitor the reactions. Incomplete couplings and deprotections were repeated. Liquid-phase reactions were monitored by LC-MS using an Agilent 1260 Infinity II LC/MSD XT system (Amsteelven, The Netherlands) equipped with an Agilent Infinity Lab Poroshell 120 EC-C18 column (3 × 100 mm, 2.7 µm) and the Agilent OpenLab CDS Chemstation software. The mobile phase consisted of Solvent A (0.1% formic acid (FA) in water) and Solvent B (0.1% FA in acetonitrile (ACN)). The following LC gradient was used for all analyses: 0–5 min, 5–100% B; 5–8 min, 100% B. The flow rate was 0.5 mL/min, and the eluents were monitored at either 220 nm, 254 nm or 280 nm. Electrospray ionization in positive mode was used to confirm the identity of the obtained products. Purification was performed in an Agilent 1290 Infinity II HPLC system using an Agilent 5 Prep C18 column (50 × 21.2 mm, 5 µm). The mobile phase consisted of solvents A and B at a flow rate of 10 mL/min. Chromatograms were recorded at either 220 or 254 nm. For purification, the following methods were used as specified: Method 1: 0–7 min, 22% B; 7–8 min, 22–100% B; 8–10 min, 100% B; and Method 2: 0–8 min, 5–100% B; 8–10 min, 100% B.

#### 4.1.2. Radiochemistry

[^111^In]InCl_3_ was purchased from Curium Netherlands BV (Petten, The Netherlands). [^177^Lu]LuCl_3_ (LuMark^®^) was purchased from IDB Holland (Baarle-Nassau, The Netherlands). Radioactivity was measured with a PerkinElmer Wizard 2 γ-counter (Groningen, The Netherlands). Instant thin-layer chromatography iTLC-SG plates (Agilent; Folsom, CA, USA) were analyzed by a bSCAN radio-chromatography scanner (Brightspec; Antwerp, Belgium). A Waters Alliance e2695 system (Etten-Leur, The Netherlands) was used to perform radio-HPLC. The system was equipped with a 2998 diode array (PDA) detector for UV detection and a NaI(Tl) Scionix crystal (Bunnik, The Netherlands) connected to a Canberra Osprey multichannel analyzer and signal amplifier (Zellik, Belgium) for detection of the radioactive signal. The software Empower 3 was used to analyze the chromatograms. The reversed-phase analytical Gemini C_18_ column (250 × 4.6 mm, 5 µm) from Phenomenex (Torrance, CA, USA) was used for all analyses. The mobile phase consisted of Solvent C (0.1% trifluoroacetic acid (TFA) in H_2_O) and Solvent D (0.1% TFA in ACN). Analyses were performed at a flow rate of 1 mL/min using the following gradient of solvents C and D: 0–3 min, 5% D; 3–23 min, 5–100% D; 23–27 min, 100% D. HPLC eluates were monitored for their UV absorbance at 254 nm.

### 4.2. Chemical Synthesis

#### 4.2.1. Synthesis of EuK(Ahx-Sta-Phe-Asp-Lys(C(O)-(CH_2_)_2_-SH)-DOTA) (**1**)

The PSMA moiety Glu-urea-Lys (EuK) was synthesized following the protocol of Derks et al. (scale of 0.1 mmol) [47]. To the resin-bound EuK moiety, a solution of Fmoc-6-Ahx-OH (141 mg, 0.40 mmol, 4 equiv.) in dimethylformamide (DMF; 2 mL) with 2-(1H-benzotriazol-1-yl)-1,1,3,3-tetramethyluronium hexafluorophosphate (HBTU; 148 mg, 0.39 mmol, 3.9 equiv.), ethyl cyano(hydroxyimino)acetate (Oxyma Pure; 57 mg, 0.40 mmol, 4 equiv.) and *N*,*N*-Diisopropylethylamine (DIPEA; 174 µL, 1.00 mmol, 10 equiv.) was added to the resin and agitated for 45 min. The resin was washed with DMF (5×), and the Fmoc group was deprotected by treatment with a solution of 20% piperidine in DMF (2 mL) for 3 × 5 min. The resin was washed with DMF (5×) before further elongation with Fmoc-*3S*,*4S*-Sta-OH (4 equiv.), Fmoc-L-Phe-OH (4 equiv.), Fmoc-Asp(*t*Bu)-OH (4 equiv.), ivDde-Lys(Fmoc)-OH (4 equiv.) and 3-(tritylthio)propionic acid (4 equiv.) was carried out as previously described. The ivDde group was deprotected with a solution of 5% hydrazine in DMF (2 mL) for 2 × 15 min. The resin was washed with DMF (5×), and a solution of DOTA(*t*Bu)_3_ ester (115 mg, 0.20 mmol, 2 equiv.), benzotriazol-1-yloxytripyrrolidinophosphonium hexafluorophosphate (PyBOP; 104 mg, 0.20 mmol, 2 equiv.) and DIPEA (70 µL, 0.40 mmol, 4 equiv.) in DMF (2 mL) was added to the resin. The mixture was shaken overnight. The beads were washed with DMF (5×) and dried. The peptide was cleaved from the solid support using 2 mL of a solution of TFA/triisopropylsilane(TIS)/H_2_O (*v*:*v*:*v* 95:2.5:2.5) for 2 h at rt. The compound was precipitated with ice-cold diethyl ether, and an additional deprotection step was performed by dissolving the crude product in 2 mL of the cleavage cocktail. Progress of the reaction was monitored by LC-MS. Once the deprotection was complete, the compound was purified by preparative HPLC (Method 1; *t_R_* = 3.76 min) to yield **1** as a white solid (32.1 mg, 22%). LC-MS (tR = 3.65 min, purity > 99%). ESI-MS *m*/*z*: calc’d for C64H103N13O23S 1453.70; found 1454.70 [M+H]+.

#### 4.2.2. Synthesis of EuK(Ahx-Sta-Phe-Asp-Lys(C(O)-(CH_2_)_2_-S-S-Py)-DOTA) (**2**)

**1** (10.0 mg, 6.8 µmol, 1 equiv.) was solubilized in DMF (250 µL) and acetic acid (50 µL). 2,2-dipyridyldisulfide (6.0 mg, 27.2. µmol, 4 equiv.) was added to the solution, and the mixture was stirred at rt for 15 min. Aqueous sodium acetate (500 µL, 0.2 M) was added dropwise to the solution over 5 min, and the mixture was stirred for an additional 30 min [30]. LC-MS confirmed product formation and reaction completion. The solvents were removed under vacuum, and the crude residue was redissolved in ACN/H_2_O (*v*:*v* 1:1). HPLC purification (Method 2; tR = 3.76 min) provided 2 as a white solid (7.8 mg, 73%). LC-MS (tR = 3.94 min, purity > 99%). ESI-MS *m*/*z*: calc’d for C69H106N14O23S2 1563.80; found 1564.60 [M+H]+.

#### 4.2.3. Synthesis of EuK(Ahx-Sta-Phe-Asp-Lys(C(O)-(CH_2_)_2_-S-S-DM1)-DOTA) (ePSMA-DM1; (3S,7S,22S,23S,26S,29S)-26-Benzyl-29-((S)-6-(3-((3-(((S)-1-(((14S,16S,32S,33S, 2R,4S,10E,12E,14R)-86-Chloro-14-Hydroxy-85,14-Dimethoxy-33,2,7,10-Tetramethyl-12,6-Dioxo-7-Aza-1(6,4)-Oxazinana-3(2,3)-Oxirana-8(1,3)-Benzenacyclotetradecaphane-10,12-Dien-4-yl)Oxy)-1-Oxopropan-2-yl)(Methyl)Amino)-3-Oxopropyl)Disulfaneyl)Propanamido)-2-(2-(4,7,10-Tris(Carboxymethyl)-1,4,7,10-Tetraazacyclododecan-1-Yl)Acetamido)Hexanamido)-22-Hydroxy-23-Isobutyl-5,13,20,25,28-Pentaoxo-4,6,12,19,24,27-Hexaazatriacontane-1,3,7,30-Tetracarboxylic Acid)

The solution of **2** (3.1 mg, 2.0 µmol, 1 equiv.) was dissolved in DMF (100 µL). DM1 (1.5 mg, 3.0 µmol, 1.5 equiv.) and triethylamine (0.84 µL, 6.0 µmol, 3.0 equiv.) were added to the solution of **2**. The reaction mixture was stirred at rt for 15 min. Reaction completion was confirmed by LC-MS. The crude product was purified by HPLC (Method 2; *t_R_* = 4.45 min) to result in ePSMA-DM1 as a white solid (2.9 mg, 66%). LC-MS (tR = 4.35 min, purity > 99%). ESI-MS *m*/*z*: calc’d for C99H149ClN16O33S2 2190.93; found 2191.70 [M+H]+.

### 4.3. Radiochemistry

#### 4.3.1. Radiolabeling with [^111^In]InCl_3_

Radiolabeling was performed in a solution containing sodium acetate (1 μL, 2.5 M), ascorbic and gentisic acids (10 μL, 50 mM), L-methionine (10 μL, 50 mM) and Milli-Q water (final volume: 140 μL) [48,49]. Concentration of the precursor in solution was determined via titration [50]. ePSMA-DM1 (1 nmol) and [^111^In]InCl_3_ (50 MBq, 370 MBq/mL) were added, and the reaction mixture was heated at 90 °C for 5 min. Quality control was performed using iTLC-SG plates eluted with a solution of sodium citrate (0.1 M, pH 5.0). Diethylenetriaminepentaacetic acid (DTPA, 5 μL, 4 mM) was added to complex-free In-111 before its injection for analytical radio-HPLC.

#### 4.3.2. Radiolabeling with [^177^Lu]LuCl_3_

Radiolabeling of ePSMA-DM1 (1 nmol) with Lu-177 (50 MBq) was performed according to the protocol described above. 

#### 4.3.3. Stability in Phosphate Buffered Saline (PBS)

[^111^In]In-ePSMA-DM1 (~10 MBq, 20 µL) was mixed with 80 μL of PBS (0.01 M, pH 7.4) and incubated at 37 °C for up to 24 h. A sample of the PBS solution was loaded directly onto the radio-HPLC at 1, 4 and 24 h post incubation. 

#### 4.3.4. Stability in Mouse Serum

[^111^In]In-ePSMA-DM1 (~30 MBq, 70 µL) was mixed with 330 μL of mouse serum and incubated at 37 °C for up to 24 h. To a separate vial containing 50 µL of acetonitrile, 50 μL of the mouse serum solution was added after 1, 4 and 24 h of incubation. The sample was centrifuged at 5000× *g* for 20 min. The supernatant was analyzed by radio-HPLC.

#### 4.3.5. Determination of LogD_7.4_ Value

The distribution coefficients (LogD_7.4_ values) were determined by the shake-flask method, in which 2 μL (~0.5 MBq) of [^111^In]In-ePSMA-DM1 was added to an Eppendorf tube containing 1 mL of PBS (0.01 M, pH 7.4)/n-octanol (*v*/*v*, 1:1). After vigorous vortexing, the solution was centrifuged at 2500 g for 3 min for phase separation. The n-octanol phase was separated from the PBS phase into a new Eppendorf tube and centrifuged for 15 min. Samples (3 × 10 μL) of the two phases were taken out and measured in the γ-counter. LogD_7.4_ values were calculated by using the following equation: LogD_7.4_ = log [(counts in octanol phase)/(counts in aqueous phase)] and the experiment was performed in triplicate [51]. 

### 4.4. Biological Assays

#### 4.4.1. NAALADase Assay

Recombinant human PSMA (rhPSMA; R & D systems, Abingdon, UK) was diluted in assay buffer (50 mM HEPES, 0.1 M NaCl, pH 7.5) to 0.4 μg/mL. The substrate Ac-Asp-Glu (40 μM) was mixed with ePSMA-DM1 or with a reference (PSMA-617) at concentrations ranging from 10^−6^ to 10^−13^ M in a final volume of 12.5 μL assay buffer. The mixtures were combined with 12.5 μL of the rhPSMA solution and incubated for 1 h at 37 °C in 384-well black polystyrene microplates (ThermoFisher, Bleiswijk, The Netherlands). A volume of 25 μL of a working solution of the Amplex Red glutamic acid kit (ThermoFisher, Bleiswijk, The Netherlands) was added and incubated at 37 °C for 30 min. Release of glutamate was measured by fluorescence with a HIDEX Sense Optical System (Goedereede, The Netherlands) set at 535 nm (excitation) and 590 nm (emission). Data were normalized, and one-site total binding regression was applied for analysis with GraphPad Prism v9.0 (GraphPad Software, San Diego, CA, USA). 

#### 4.4.2. GSH Stability

The protocol was adapted from the literature [30]. A 5 mM solution of L-glutathione was prepared in PBS, and 990 μL aliquots were dispensed into Eppendorf tubes. To each tube, 10 μL of a solution of ePSMA-DM1 (1 mM) in MeOH was added, and the mixtures were incubated at 37 °C. Then 50 μL aliquots of the solution were taken at 10 min, 30 min, 1, 2, 4 and 6 h and analyzed by LC-MS. The experiments were performed in triplicate.

#### 4.4.3. Cell Culture

LS174T human colon carcinoma cells transfected with human PSMA were cultured in RPMI-1640 media supplemented with 2 mM glutamine, 10% fetal bovine serum (FBS) and 0.3 mg/mL G418 (Geneticin, Sigma Aldrich, Burlington, MA, USA). The cells were provided by Dr. Sandra Heskamp (Radboud UMC, Nijmegen, The Netherlands). 

#### 4.4.4. Cytotoxicity Assay

A resazurin in vitro toxicology assay kit was used to determine cytotoxicity. Briefly, 5 × 10^3^ LS174T cells were seeded in a volume of 100 µL per well in 96-well plates and incubated at 37 °C with 5% CO_2_ for 24 h before the experiment. After incubation, the media were removed, and the wells were washed once with PBS. Then, ePSMA-DM1 at the concentrations of 1, 10, 100 and 1000 nM and the positive control DM1 (1000 nM) were prepared in culture media (RPMI-1640 media supplemented with 2 mM glutamine, 10% fetal bovine serum (FBS) and 0.3 mg/mL G418). Media and PSMA-617 (100 nM) were used as negative controls. Cells were incubated with 100 µL of the tested compounds for 48 and 72 h at 37 °C with 5% CO_2_. After incubation, media were removed, and each well was washed once with PBS. Next, 100 µL of 10% resazurin solution (TOX8, Sigma-Aldrich) in culture media was added to each well and incubated for 2.5 h. Fluorescence was measured using the HIDEX Sense Optical System with excitation at 544 nm and emission at 590 nm. Data were analyzed using GraphPad Prism 9.0 and expressed as cell viability (%). The experiments were performed in triplicate.

#### 4.4.5. Internalization and Cell Uptake Assay

Cells (1.2 × 10^6^ cells/well) were seeded and cultured to 80% confluency for 24 h prior to experiments. 10^−9^ M solutions of [^111^In]In-PSMA-617 and [^111^In]In-ePSMA-DM1 were prepared in internalization media (RPMI, 20 mM HEPES, 1% BSA, pH 7.4). Both compounds were also evaluated with a block of 50-fold excess non-labelled PSMA-617. The media were removed from each well, which were then rinsed twice with PBS. A total of 2 mL of each radioactive compound were added to the wells and incubated for 2 h at 37 °C. For the internalization assay, the media containing radioactive compound was removed, and each well was washed twice with 1 mL cold PBS. A total of 1 mL of Glycine buffer (50 mM glycine, 100 mM NaCl, pH 2.8) was added immediately and incubated for 10 min at rt. The glycine wash (membrane-bound fraction) was then collected into counting tubes. An extra wash was performed and collected in the same tubes. A total of 1 mL of NaOH (1 M) was added to each tube and incubated for 15 min at rt. The lysate (internalized fraction) was then collected in separate counting tubes, together with one extra NaOH wash. Cells were counted, and data were normalized to 1,000,000 cells and percentage added dose (%). The experiments were performed in triplicate.

### 4.5. Animal Studies

#### 4.5.1. Mouse Model

Animal experiments were performed using male BALB/c nude mice (8–10 weeks old; Janvier Labs, Le Genest-Saint-Isle, France). Animals were housed in individually ventilated cages (Blue line IVC, 2 mice per cage) under sterile standard conditions with cage enrichments present and free access to animal chow (Sniff GmbH, Soest, Germany) and water. They were acclimated for 1 week prior to experiments. Mice were subcutaneously inoculated with 4.0 × 10^6^ LS174T cells in the right flank diluted in 100 μL of cell suspension 7 days after tumor cell inoculation. The average tumor volume size per group (n = 4) was of 206.2 ± 7.9 mm^3^ (average weight of 105.8 ± 7.3 mg) after non-blinded grouping to enable homogenous tumor size distribution. [^111^In]In-ePSMA-DM1 was injected intravenously via the tail vein. All experiments were approved by the institutional Animal Welfare Committee and were conducted in accordance to the guidelines of the Revised Dutch Act on Animal Experimentation (WOD). 

#### 4.5.2. Ex Vivo Biodistribution

Mice were intravenously injected with ~1 nmol of [^111^In]In-ePSMA-DM1 (5 MBq). Mice (n = 4/timepoint) were euthanized by cervical dislocation at 1, 4 and 24 h p.i. A block group (co-injection of 50 nmol of PSMA-617) was included, and mice were euthanized at 4 h p.i. Organs (blood, heart, lungs, liver, spleen, stomach, intestines, pancreas, kidneys, muscle, skin, bone and prostate) and the tumors were collected in pre-weighed tubes, weighed after organ collection, and measured in the γ-counter, for which the calibration factor was determined by measuring different known activities of In-111. Activity was corrected for decay, and uptake was expressed as percentage of the injected dose per gram of the tissue (% ID/g). The biological half-lives in tissues of [^111^In]In-ePSMA-DM1 were determined using a one-phase decay algorithm in GraphPad Prism 9.0 (GraphPad Software, Boston, MA, USA).

#### 4.5.3. SPECT-CT Imaging

Mice were intravenously injected with ~1 nmol of [^111^In]In-ePSMA-DM1 (20 MBq) and imaged at 1 and 24 h p.i. in a dedicated small-animal PET/SPECT/CT scanner (VECTor^5^CT scanner, MILabs B.V., Utrecht, The Netherlands). Mice were placed on a heated bed under 2% isoflurane/O_2_ anesthesia during the scans. A high-sensitivity pinhole collimator (XXUHS-M, 3.00 mm pinhole diameter) was used, and whole-body SPECT images (transaxial field of view, 54 mm) were acquired over 30 min using a spiral scan in normal scan mode with in list-mode acquisition. This was followed by a whole-body CT scan within 5 min (settings: full angle scan, angle step 0.75 degrees, normal scan mode, 50 kV tube voltage, 0.21 mA tube current, 500 µm aluminum filter). For reconstruction of the SPECT images, the similarity-regulated SROSEM method and MLMN method (MILabs Rec 12.00 software) were employed, performing 9 and 128 iterations, respectively, at 0.8 mm^3^ resolution, using 173 keV ± 10% and 247 keV ± 10% energy windows for In-111. Two adjacent background windows per photo peak were selected for triple-energy window scatter and for crosstalk correction. Post-filtering of reconstructed volumes of SPECT scans was performed with an isotropic 3-dimensional Gaussian filter with 1 mm full width at half-maximum. The CT and registered, attenuation-corrected SPECT images were analyzed using IMALYTICS Preclinical 3.0 (Gremse-IT GmbH, Aachen, Germany).

#### 4.5.4. Statistical Analysis

GraphPad Prism 9.0 was used for all statistical analyses. Outliers were tested using a Grubbs outlier test, and normality was tested using the Shapiro–Wilk normality test. Significant differences for the uptake and internalization, cytotoxicity and ex vivo biodistribution were evaluated using an unpaired *t*-test or a one-way ANOVA test. A Kruskal–Wallis test was used when data was not normal. The difference was considered statistically significant if the *p*-value was <0.05. *p*-values smaller than 0.05 (*p* < 0.05), *p* < 0.01, *p* < 0.001 and *p* < 0.0001 are indicated with one (*), two (**), three (***) or four (****) asterisks, respectively. Data are reported as average ± standard deviation (SD). 

## 5. Conclusions

We reported the synthesis, radiolabeling and preliminary biological evaluation of ePSMA-DM1, a promising theranostic small-molecule drug conjugate (T-SMDC) targeting PSMA. The compound was synthesized with a good global chemical yield (11%) and demonstrated a high radiochemical purity and yield (>95%), as well as good stability in PBS and serum, when radiolabeled with both In-111 and Lu-177 (>80% up to 24 h). The conjugate showed good binding to PSMA (IC_50_ = 45.3 ± 1.84 nM) and PSMA-specific uptake with 48% corresponding to the internalized fraction. Cytotoxicity assays with the non-radiolabeled compound showed a 85% reduction in cell viability 72 h post incubation at doses of 100 and 1000 nM. The in vivo and ex vivo evaluations of [^111^In]In-ePSMA-DM1 showed a tumor uptake of 2.39 ± 0.29% ID/g at 1 h p.i., and the compound remained in the tumor up to 24 h p.i. The tumor could be visualized by SPECT/CT at 1 h p.i.; however, the signal was faint. Uptake in off-target organs was observed, both in vivo and ex vivo, suggesting that the T-SMDC might act through a non-PSMA-specific mechanism. Before conducting therapeutic experiments, the mechanisms of the uptake of ePSMA-DM1 need to be elucidated in order to minimize toxicity to off-target organs.

## Figures and Tables

**Table 1:** Radiochemical data obtained for [^111^In]In-ePSMA-DM1 and [^177^Lu]Lu-ePSMA-DM1.

Compound	RCY (iTLC, %)	RCP (HPLC, %)	LogD_7.4_	Stability in PBS (%) ^1^	Stability in Mouse Serum (%) ^1^
[^111^In]In-ePSMA-DM1	97.2 ± 1.3 ^2^	92.6 ± 2.2 ^2^	−2.47 ± 0.11 ^2^	90.9	82.9
[^177^Lu]Lu-ePSMA-DM1	96.3	94.8	-	81.4	91.2

^1^ Values refer to the timepoint of 24 h post incubation. ^2^ Experiments were performed in triplicate.

## Data Availability

The data present in this study are available in the body of the manuscript and in the Supplementary Materials.

## Supplementary Materials

The following supporting information can be downloaded at: https://www.mdpi.com/article/10.3390/ph16081072/s1, Figure S1: Characterization of compounds **1**, **2** and ePSMA-DM1; Figures S2–S4: Radiochemistry of [^111^In]In-ePSMA-DM1; Figures S5 and S6: Radiochemistry of [^177^Lu]Lu-ePSMA-DM1; Figure S7: NAALADase assay of ePSMA-DM1 and the reference PSMA-617; Table S1: Ex vivo biodistribution data of [^111^In]In-ePSMA-DM1.

## Abbreviations

AD: added dose; ADC: antibody–drug conjugate; DIPEA: *N*,*N*-diisopropylethylamine; DMF: dimethylformamide; DOTA: 2,2′,2″,2‴-(1,4,7,10-tetraazacyclododecane-1,4,7,10-tetrayl)tetraacetic acid; DUPA: 2-[3-(1,3-dicarboxypropyl)ureido]pentanedioic acid; Et_3_N: triethylamine; EuK: glutamate-urea-lysine; FDA: U.S. Food and Drug Administration; GSH: glutathione; HBTU: 2-(1H-benzotriazol-1-yl)-1,1,3,3-tetramethyluronium hexafluorophosphate; IC_50_: half maximal inhibitory concentration; iTLC: instant thin-layer chromatography; HPLC: high-performance liquid chromatography; LC-MS: liquid chromatography–mass spectrometry; mCRPC: metastatic castration-resistant prostate cancer; MIP: maximal intensity projection; PBS: phosphate buffered saline; PyBOP: benzotriazol-1-yloxytripyrrolidinophosphonium hexafluorophosphate; PSMA: prostates-specific membrane antigen; RCP: radiochemical purity; RCY: radiochemical yield; rhPSMA: recombinant human PSMA; RLT: radioligand therapy; SMDC: small-molecule drug conjugate; SPECT/CT: single photon emission computed tomography/computed tomography; SPPS: solid-phase peptide synthesis; TFA: trifluoroacetic acid; TIS: triisopropylsilane; T-SMDC: theranostic small-molecule drug conjugate.

## References

[1] Cornford P., van den Bergh R.C.N., Briers E., Van den Broeck T., Cumberbatch M.G., De Santis M., Fanti S., Fossati N., Gandaglia G., Gillessen S. (2021). EAU-EANM-ESTRO-ESUR-SIOG Guidelines on Prostate Cancer. Part II—2020 Update: Treatment of Relapsing and Metastatic Prostate Cancer [Formula Presented]. Eur. Urol..

[2] Zhong L., Li Y., Xiong L., Wang W., Wu M., Yuan T., Yang W., Tian C., Miao Z., Wang T. (2021). Small Molecules in Targeted Cancer Therapy: Advances, Challenges, and Future Perspectives. Signal Transduct. Target. Ther..

[3] Debnath S., Zhou N., McLaughlin M., Rice S., Pillai A.K., Hao G., Sun X. (2022). PSMA-Targeting Imaging and Theranostic Agents—Current Status and Future Perspective. Int. J. Mol. Sci..

[4] Wright G.L., Haley C., Beckett M.L., Schellhammer P.F. (1995). Expression of Prostate-Specific Membrane Antigen in Normal, Benign, and Malignant Prostate Tissues. Urol. Oncol. Semin. Orig. Investig..

[5] Sgouros G., Bodei L., McDevitt M.R., Nedrow J.R. (2020). Radiopharmaceutical Therapy in Cancer: Clinical Advances and Challenges. Nat. Rev. Drug Discov..

[6] Duan H., Iagaru A., Aparici C.M. (2022). Radiotheranostics—Precision Medicine in Nuclear Medicine and Molecular Imaging. Nanotheranostics.

[7] Hofman M.S., Emmett L., Sandhu S., Iravani A., Joshua A.M., Goh J.C., Pattison D.A., Tan T.H., Kirkwood I.D., Ng S. (2021). [^177^Lu]Lu-PSMA-617 versus Cabazitaxel in Patients with Metastatic Castration-Resistant Prostate Cancer (TheraP): A Randomised, Open-Label, Phase 2 Trial. Lancet.

[8] Pathmanandavel S., Crumbaker M., Yam A.O., Nguyen A., Rofe C., Hovey E., Gedye C., Kwan E.M., Hauser C., Azad A.A. (2022). ^177^Lu-PSMA-617 and Idronoxil in Men with End-Stage Metastatic Castration-Resistant Prostate Cancer (LuPIN): Patient Outcomes and Predictors of Treatment Response in a Phase I/II Trial. J. Nucl. Med..

[9] Suman S., Parghane R.V., Joshi A., Prabhash K., Talole S., Basu S. (2021). Combined ^177^Lu-PSMA-617 PRLT and Abiraterone Acetate versus ^177^Lu-PSMA-617 PRLT Monotherapy in Metastatic Castration-Resistant Prostate Cancer: An Observational Study Comparing the Response and Durability. Prostate.

[10] Emmett L., Subramaniam S., Joshua A.M., Crumbaker M., Martin A., Zhang A.Y., Rana N., Langford A., Mitchell J., Yip S. (2021). ENZA-p Trial Protocol: A Randomized Phase II Trial Using Prostate-Specific Membrane Antigen as a Therapeutic Target and Prognostic Indicator in Men with Metastatic Castration-Resistant Prostate Cancer Treated with Enzalutamide (ANZUP 1901). BJU Int..

[11] Sandhu S., Joshua A.M., Emmett L., Spain L.A., Horvath L., Crumbaker M., Anton A., Wallace R., Pasam A., Bressel M. (2022). PRINCE: Phase I Trial of ^177^Lu-PSMA-617 in Combination with Pembrolizumab in Patients with Metastatic Castration-Resistant Prostate Cancer (MCRPC). J. Clin. Oncol..

[12] Sandhu S., Guo C., Hofman M.S. (2021). Radionuclide Therapy in Prostate Cancer: From Standalone to Combination PSMA Theranostics. J. Nucl. Med..

[13] Suman S.K., Subramanian S., Mukherjee A. (2021). Combination Radionuclide Therapy: A New Paradigm. Nucl. Med. Biol..

[14] Fu Z., Li S., Han S., Shi C., Zhang Y. (2022). Antibody Drug Conjugate: The “Biological Missile” for Targeted Cancer Therapy. Signal Transduct. Target. Ther..

[15] Milowsky M.I., Galsky M.D., Morris M.J., Crona D.J., George D.J., Dreicer R., Tse K., Petruck J., Webb I.J., Bander N.H. (2016). Phase 1/2 Multiple Ascending Dose Trial of the Prostate-Specific Membrane Antigen-Targeted Antibody Drug Conjugate MLN2704 in Metastatic Castration-Resistant Prostate Cancer. Urol. Oncol. Semin. Orig. Investig..

[16] Petrylak D.P., Kantoff P., Vogelzang N.J., Mega A., Fleming M.T., Stephenson J.J., Frank R., Shore N.D., Dreicer R., McClay E.F. (2019). Phase 1 Study of PSMA ADC, an Antibody-Drug Conjugate Targeting Prostate-Specific Membrane Antigen, in Chemotherapy-Refractory Prostate Cancer. Prostate.

[17] Zhuang C., Guan X., Ma H., Cong H., Zhang W., Miao Z. (2019). Small Molecule-Drug Conjugates: A Novel Strategy for Cancer-Targeted Treatment. Eur. J. Med. Chem..

[18] Olatunji F.P., Pun M., Herman J.W., Romero O., Maniatopoulos M., Latoche J.D., Parise R.A., Guo J., Beumer J.H., Anderson C.J. (2022). Modular Smart Molecules for PSMA-Targeted Chemotherapy. Mol. Cancer Ther..

[19] Boinapally S., Ahn H.-H., Cheng B., Brummet M., Nam H., Gabrielson K.L., Banerjee S.R., Minn I., Pomper M.G. (2021). A Prostate-Specific Membrane Antigen (PSMA)-Targeted Prodrug with a Favorable in Vivo Toxicity Profile. Sci. Rep..

[20] Wang X., Shirke A., Walker E., Sun R., Ramamurthy G., Wang J., Shan L., Mangadlao J., Dong Z., Li J. (2021). Small Molecule-Based Prodrug Targeting Prostate Specific Membrane Antigen for the Treatment of, Prostate Cancer. Cancers.

[21] Kularatne S.A., Wang K., Santhapuram H.K.R., Low P.S. (2009). Prostate-Specific Membrane Antigen Targeted Imaging and Therapy of Prostate Cancer Using a PSMA Inhibitor as a Homing Ligand. Mol. Pharm..

[22] Leamon C.P., Reddy J.A., Bloomfield A., Dorton R., Nelson M., Vetzel M., Kleindl P., Hahn S., Wang K., Vlahov I.R. (2019). Prostate-Specific Membrane Antigen-Specific Antitumor Activity of a Self-Immolative Tubulysin Conjugate. Bioconjug. Chem..

[23] Roy J., Nguyen T.X., Kanduluru A.K., Venkatesh C., Lv W., Reddy P.V.N., Low P.S., Cushman M. (2015). DUPA Conjugation of a Cytotoxic Indenoisoquinoline Topoisomerase i Inhibitor for Selective Prostate Cancer Cell Targeting. J. Med. Chem..

[24] Machulkin A.E., Skvortsov D.A., Ivanenkov Y.A., Ber A.P., Kavalchuk M.V., Aladinskaya A.V., Uspenskaya A.A., Shafikov R.R., Plotnikova E.A., Yakubovskaya R.I. (2019). Synthesis and Biological Evaluation of PSMA-Targeting Paclitaxel Conjugates. Bioorg. Med. Chem. Lett..

[25] Machulkin A.E., Uspenskaya A.A., Zyk N.Y., Nimenko E.A., Ber A.P., Petrov S.A., Shafikov R.R., Skvortsov D.A., Smirnova G.B., Borisova Y.A. (2022). PSMA-Targeted Small-Molecule Docetaxel Conjugate: Synthesis and Preclinical Evaluation. Eur. J. Med. Chem..

[26] Srinivasarao M., Galliford C.V., Low P.S. (2015). Principles in the Design of Ligand-Targeted Cancer Therapeutics and Imaging Agents. Nat. Rev. Drug Discov..

[27] Choi W.G., Park R., Kim D.K., Shin Y., Cho Y.Y., Lee H.S. (2020). Mertansine Inhibits MRNA Expression and Enzyme Activities of Cytochrome P450s and Uridine 5′-Diphospho-Glucuronosyltransferases in Human Hepatocytes and Liver Microsomes. Pharmaceutics.

[28] Huang C.T., Guo X., Bařinka C., Lupold S.E., Pomper M.G., Gabrielson K., Raman V., Artemov D., Hapuarachchige S. (2020). Development of 5D3-DM1: A Novel Anti-Prostate-Specific Membrane Antigen Antibody-Drug Conjugate for PSMA-Positive Prostate Cancer Therapy. Mol. Pharm..

[29] Barok M., Joensuu H., Isola J. (2014). Trastuzumab Emtansine: Mechanisms of Action and Drug Resistance. Breast Cancer Res..

[30] White B.H., Whalen K., Kriksciukaite K., Alargova R., Au Yeung T., Bazinet P., Brockman A., Dupont M., Oller H., Lemelin C.A. (2019). Discovery of an SSTR2-Targeting Maytansinoid Conjugate (PEN-221) with Potent Activity In Vitro and In Vivo. J. Med. Chem..

[31] Kumar A., Mastren T., Wang B., Hsieh J.T., Hao G., Sun X. (2016). Design of a Small-Molecule Drug Conjugate for Prostate Cancer Targeted Theranostics. Bioconjug. Chem..

[32] Krall N., Pretto F., Decurtins W., Bernardes G.J.L., Supuran C.T., Neri D. (2014). A Small-Molecule Drug Conjugate for the Treatment of Carbonic Anhydrase IX Expressing Tumors. Angew. Chem. Int. Ed..

[33] Hillier S.M., Maresca K.P., Femia F.J., Marquis J.C., Foss C.A., Nguyen N., Zimmerman C.N., Barrett J.A., Eckelman W.C., Pomper M.G. (2009). Preclinical Evaluation of Novel Glutamate-Urea-Lysine Analogues That Target Prostate-Specific Membrane Antigen as Molecular Imaging Pharmaceuticals for Prostate Cancer. Cancer Res..

[34] Eder M., Schäfer M., Bauder-Wüst U., Hull W.E., Wängler C., Mier W., Haberkorn U., Eisenhut M. (2012). 68Ga-Complex Lipophilicity and the Targeting Property of a Urea-Based PSMA Inhibitor for PET Imaging. Bioconjug. Chem..

[35] Lv Q., Yang J., Zhang R., Yang Z., Yang Z., Wang Y., Xu Y., He Z. (2018). Prostate-Specific Membrane Antigen Targeted Therapy of Prostate Cancer Using a DUPA–Paclitaxel Conjugate. Mol. Pharm..

[36] Wu M., Huang W., Yang N., Liu Y. (2022). Learn from Antibody–Drug Conjugates: Consideration in the Future Construction of Peptide-Drug Conjugates for Cancer Therapy. Exp. Hematol. Oncol..

[37] Rana A., Bhatnagar S. (2021). Advancements in Folate Receptor Targeting for Anti-Cancer Therapy: A Small Molecule-Drug Conjugate Approach. Bioorg. Chem..

[38] Alas M., Saghaeidehkordi A., Kaur K. (2021). Peptide-Drug Conjugates with Different Linkers for Cancer Therapy. J. Med. Chem..

[39] Benešová M., Bauder-Wüst U., Schäfer M., Klika K.D., Mier W., Haberkorn U., Kopka K., Eder M. (2016). Linker Modification Strategies to Control the Prostate-Specific Membrane Antigen (PSMA)-Targeting and Pharmacokinetic Properties of DOTA-Conjugated PSMA Inhibitors. J. Med. Chem..

[40] Wüstemann T., Bauder-Wüst U., Schäfer M., Eder M., Benesova M., Leotta K., Kratochwil C., Haberkorn U., Kopka K., Mier W. (2016). Design of Internalizing PSMA-Specific Glu-Ureido-Based Radiotherapeuticals. Theranostics.

[41] Riss T.L., Moravec R.A., Niles A.L., Duellman S., Benink H.A., Worzella T.J., Minor L. (2004). Cell Viability Assays. Assay Guidance Manual.

[42] Erickson H.K., Widdison W.C., Mayo M.F., Whiteman K., Audette C., Wilhelm S.D., Singh R. (2010). Tumor Delivery and in Vivo Processing of Disulfide-Linked and Thioether-Linked Antibody-Maytansinoid Conjugates. Bioconjug. Chem..

[43] Ruigrok E.A.M., van Vliet N., Dalm S.U., de Blois E., van Gent D.C., Haeck J., de Ridder C., Stuurman D., Konijnenberg M.W., van Weerden W.M. (2021). Extensive Preclinical Evaluation of Lutetium-177-Labeled PSMA-Specific Tracers for Prostate Cancer Radionuclide Therapy. Eur. J. Nucl. Med. Mol. Imaging.

[44] Murce E., Beekman S., Spaan E., Handula M., Stuurman D., de Ridder C., Seimbille Y. (2023). Preclinical Evaluation of a PSMA-Targeting Homodimer with an Optimized Linker for Imaging of Prostate Cancer. Molecules.

[45] Felber V.B., Valentin M.A., Wester H.J. (2021). Design of PSMA Ligands with Modifications at the Inhibitor Part: An Approach to Reduce the Salivary Gland Uptake of Radiolabeled PSMA Inhibitors?. EJNMMI Radiopharm. Chem..

[46] Debnath S., Hao G., Guan B., Thapa P., Hao J., Hammers H., Sun X. (2022). Theranostic Small-Molecule Prodrug Conjugates for Targeted Delivery and Controlled Release of Toll-like Receptor 7 Agonists. Int. J. Mol. Sci..

[47] Derks Y.H.W., Rijpkema M., Amatdjais-Groenen H.I.V., Kip A., Franssen G.M., Michiel Sedelaar J.P., Somford D.M., Simons M., Laverman P., Gotthardt M. (2021). Photosensitizer-Based Multimodal PSMA-Targeting Ligands for Intraoperative Detection of Prostate Cancer. Theranostics.

[48] de Blois E., Sze Chan H., Konijnenberg M., de Zanger R., Breeman A.P.W. (2013). Effectiveness of Quenchers to Reduce Radiolysis of 111In- or 177Lu-Labelled Methionine-Containing Regulatory Peptides. Maintaining Radiochemical Purity as Measured by HPLC. Curr. Top. Med. Chem..

[49] de Zanger R.M.S., Chan H.S., Breeman W.A.P., de Blois E. (2019). Maintaining Radiochemical Purity of [^177^Lu]Lu-DOTA-PSMA-617 for PRRT by Reducing Radiolysis. J. Radioanal. Nucl. Chem..

[50] Breeman W., de Zanger R., Chan H., Blois E. (2015). Alternative Method to Determine Specific Activity of ^177^Lu by HPLC. Curr. Radiopharm..

[51] Chen K.T., Nieuwenhuizen J., Handula M., Seimbille Y. (2020). A Novel Clickable MSAP Agent for Dual Fluorescence/Nuclear Labeling of Biovectors. Org. Biomol. Chem..

