# Supplementary material for: Synthesis and Evaluation of ePSMA-DM1: A New Theranostic Small-Molecule Drug Conjugate (T-SMDC) for Prostate Cancer
Source: Pharmaceuticals (Basel). 2023 Jul 28;16(8):1072. doi: 10.3390/ph16081072 (PMC10458530; doi:10.3390/ph16081072)
Supplement: Supplementary file 1 [file pharmaceuticals-16-01072-s001.zip › pharmaceuticals-2488696-supplementary.pdf]

Supplementary Information

Synthesis and evaluation of ePSMA-DM1: a new theranostic small-molecule drug conjugate (T-SMDC) for prostate cancer

Erika Murce[1,2], Evelien Spaan[1,2], Savanne Beekman[1,2], Lilian van den Brink[1,2], Maryana Handula[1,2], Debra Stuurman[1,2], Corrina de Ridder[1,2], Simone U. Dalm[1,2] and Yann Seimbille[1,2,3].

[1]*Department of Radiology and Nuclear Medicine, University Medical Center Rotterdam, Erasmus MC, Rotterdam, The Netherlands*

[2]*Erasmus MC Cancer Institute, Rotterdam, The Netherlands*

[3]*TRIUMF, Life Sciences Division, Vancouver, Canada*

[*]*Corresponding author:* y.seimbille@erasmusmc.nl

Table of Contents

Characterization of 1, 2 and ePSMA-DM1.....2

Radiochemistry of [^{111}In]In-ePSMA-DM1.....5

Radiochemistry of [^{177}Lu]Lu-ePSMA-DM1.....7

NAALADase assay (binding affinity) of ePSMA-DM1 and the reference PSMA-617.....10

Ex vivo biodistribution of [^{111}In]In-ePSMA-DM1.....10

Characterization of 1, 2 and ePSMA-DM1

Compounds were obtained in >95% chemical purity, as verified by LC-MS. They were visualized at 254 nm and identified by the ESI-MS.

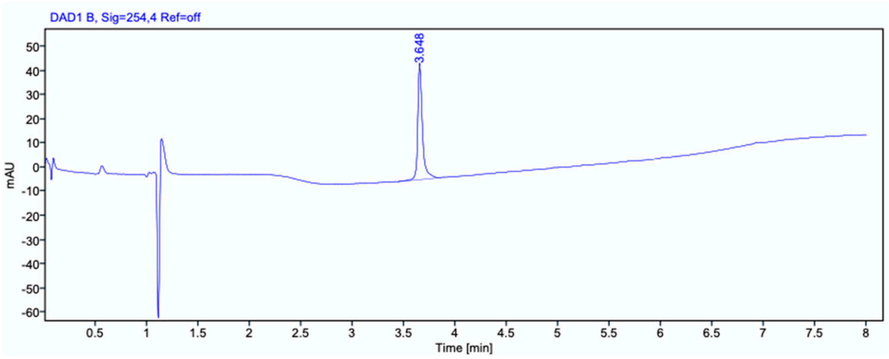

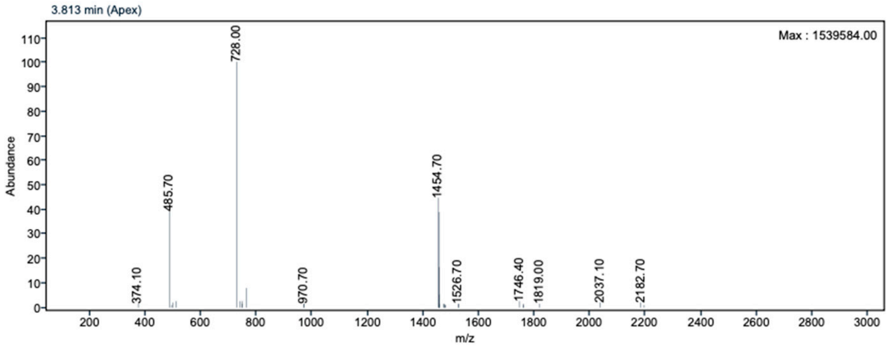

Figure S1.1: LC chromatogram and mass spectrum of **1**.

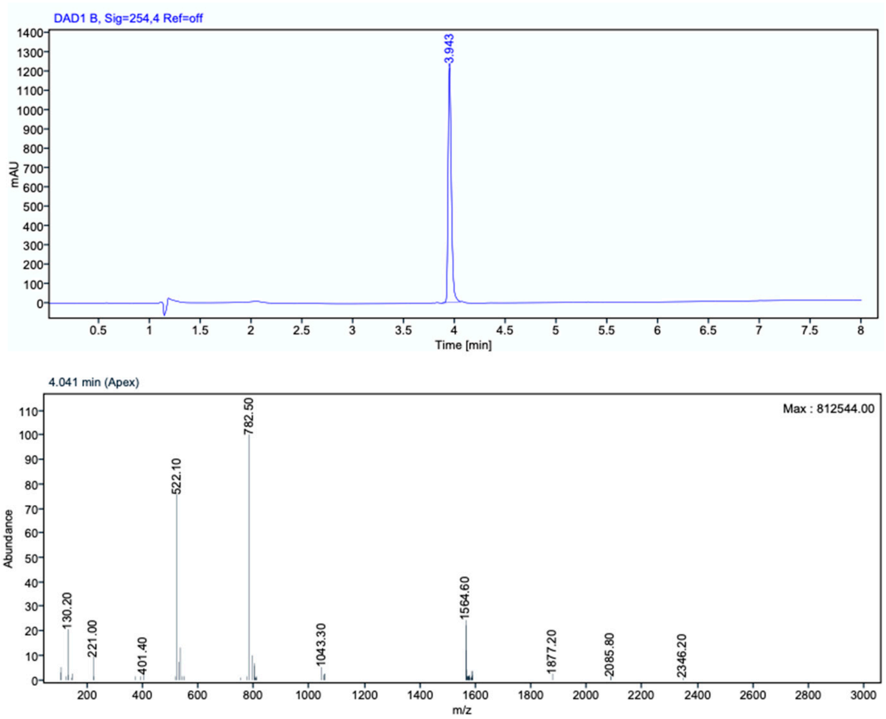

Figure S1.2: LC chromatogram and mass spectrum of **2**.

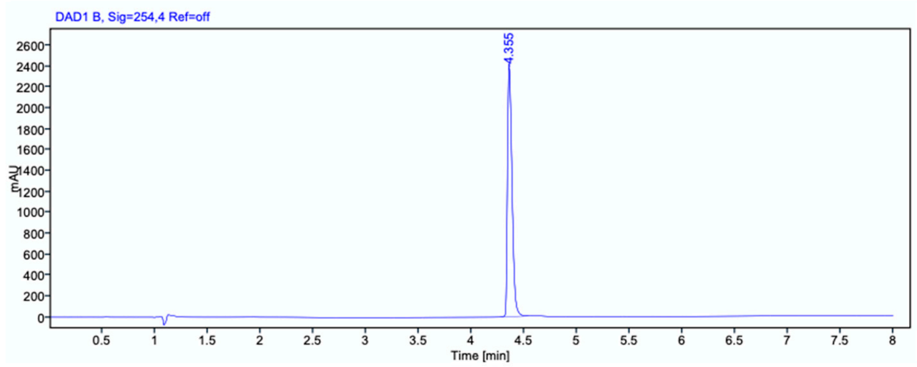

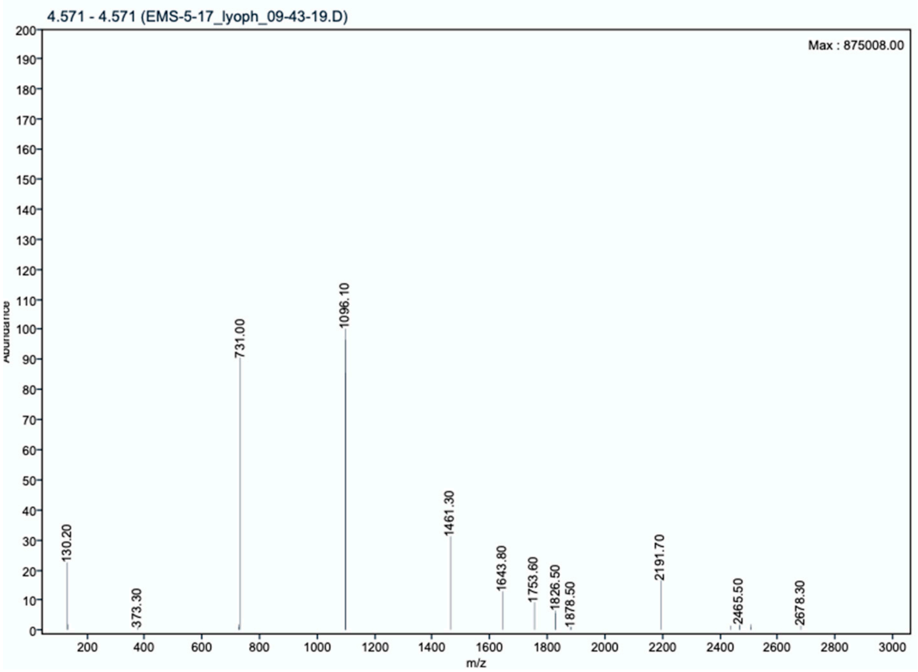

Figure S1.3: LC chromatogram and mass spectrum of **ePSMA-DM1**.

Radiochemistry of $[^{111}In]$In-ePSMA-DM1

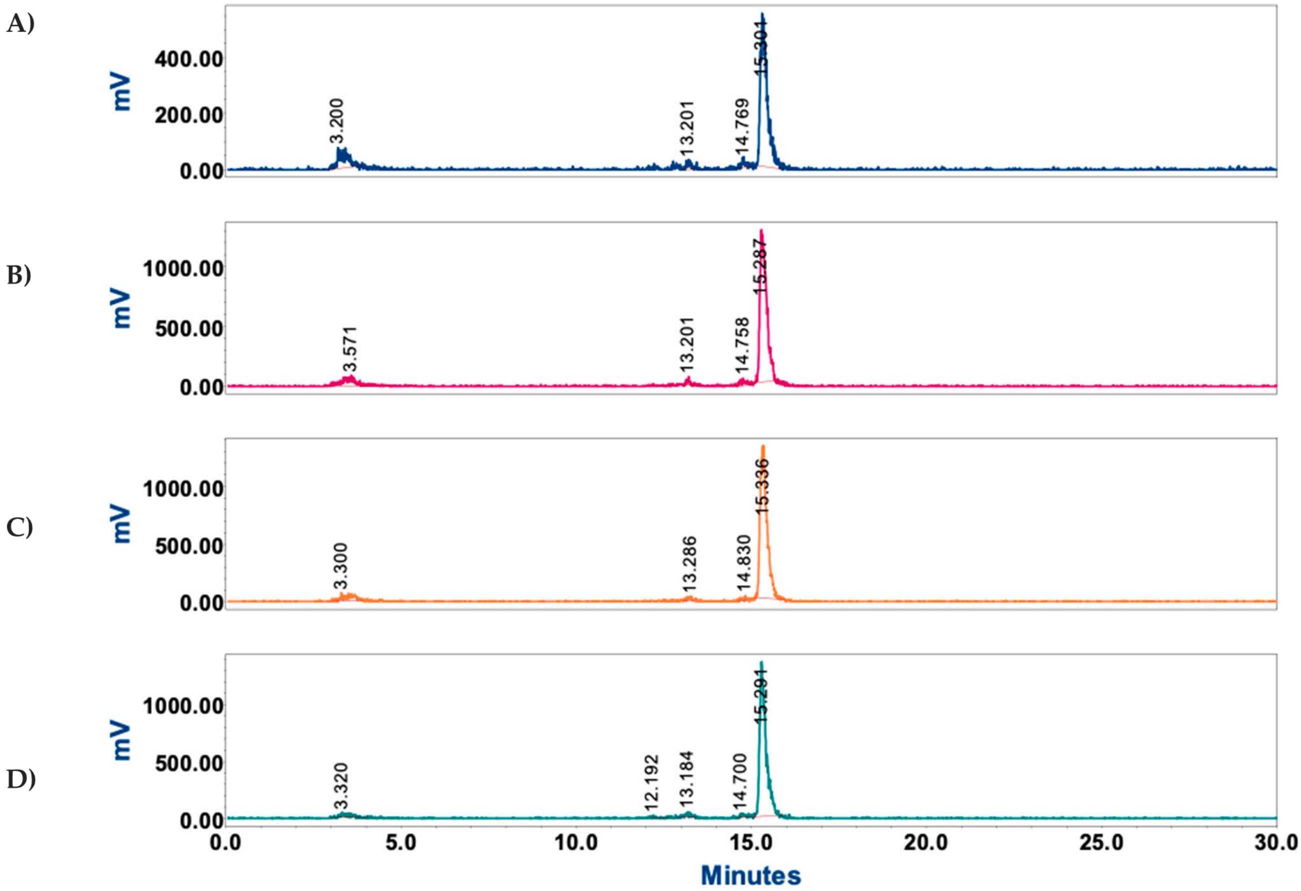

Figure S2. A) RP-HPLC chromatogram of $[^{111}In]$In-ePSMA-DM1. The peak at 15.30 minutes represents the compound $[^{111}In]$In-ePSMA-DM1**.** The peak at 3 minutes represents free ^{111}In.

B) RP-HPLC chromatogram of $[^{111}In]$In-ePSMA-DM1 incubated in PBS for 1h at 37°C.

C) RP-HPLC chromatogram of $[^{111}In]$In-ePSMA-DM1 incubated in PBS for 4h at 37°C.

D) RP-HPLC chromatogram of $[^{111}In]$In-ePSMA-DM1 incubated in PBS for 24h at 37°C.

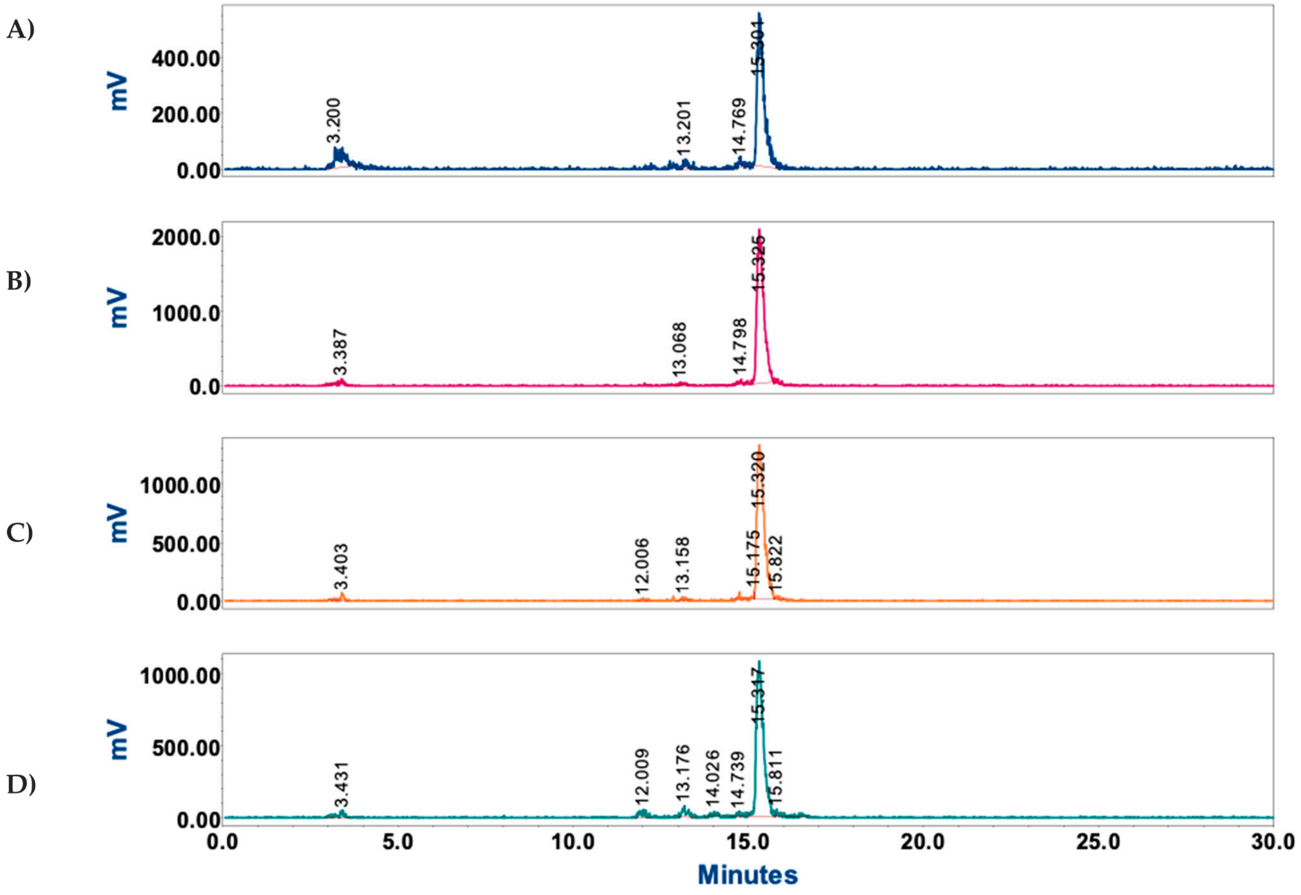

Figure S3. A) RP-HPLC chromatogram of [^{111}In]In-ePSMA-DM1. The peak at 15.30 minutes represents the compound [^{111}In]In-ePSMA-DM1. The peak at 3 minutes represents free ^{111}In.

B) RP-HPLC chromatogram of [^{111}In]In-ePSMA-DM1 incubated in mouse serum for 1h at 37°C.

C) RP-HPLC chromatogram of [^{111}In]In-ePSMA-DM1 incubated in mouse serum for 4h at 37°C.

D) RP-HPLC chromatogram of [^{111}In]In-ePSMA-DM1 incubated in mouse serum for 24h at 37°C.

A)

B)

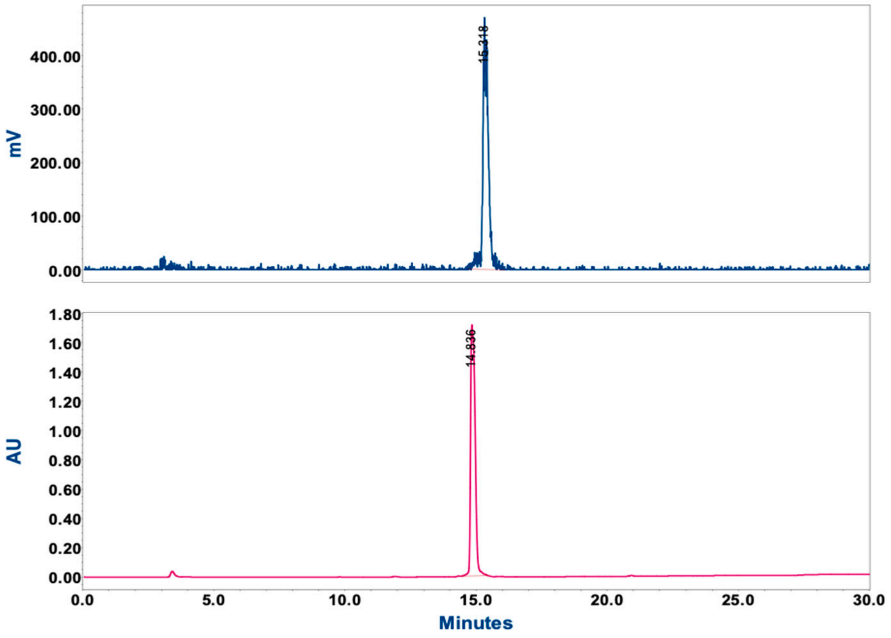

Figure S4. RP-HPLC chromatogram of the co-elution of [^{111}In]In-ePSMA-DM1 with ePSMA-DM1. 10 nmol of ePSMA-DM1 were injected in order to obtain a visible UV signal. **A**) RP-HPLC chromatogram with the radioactivity detector. The peak at 15.32 minutes represents the compound [^{111}In]In-ePSMA-DM1.

B) RP-HPLC UV-Vis chromatogram of [^{111}In]In-ePSMA-DM1, visualized at 254 nm. The peak at 14.83 minutes corresponds to ePSMA-DM1. The difference in retention time observed between [^{111}In]In-ePSMA-DM1 and ePSMA-DM1 is due to the positioning UV-Vis detector after the radioactivity detector.

Radiochemistry of [^{177}Lu]Lu-ePSMA-DM1

A)

B)

C)

D)

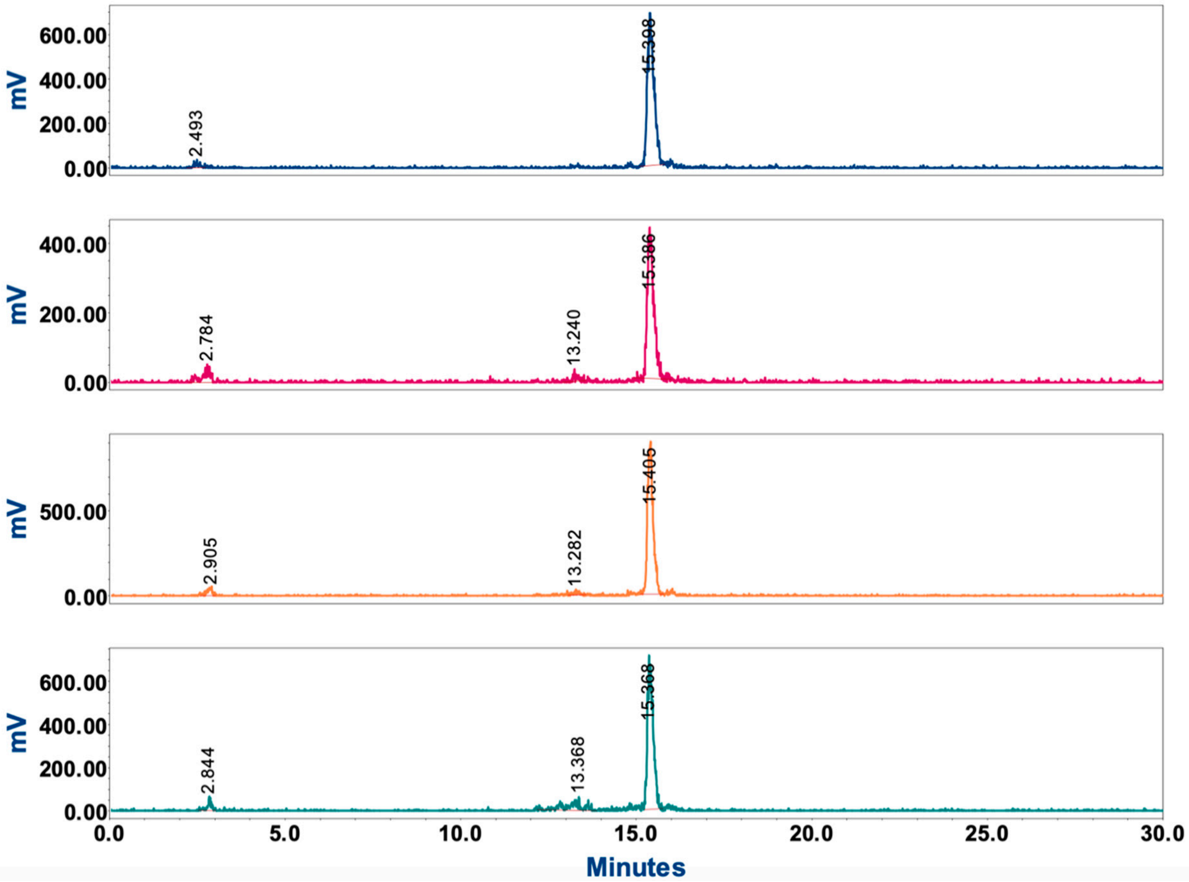

Figure S5. A) RP-HPLC chromatogram of [^{177}Lu]Lu-ePSMA-DM1. The peak at 15.308 minutes represents the compound [^{177}Lu]Lu-ePSMA-DM1. The peak at 2 minutes represents free ^{177}Lu.

B) RP-HPLC chromatogram of [^{177}Lu]Lu-ePSMA-DM1 incubated in PBS for 1h at 37°C.

C) RP-HPLC chromatogram of [^{177}Lu]Lu-ePSMA-DM1 incubated in PBS for 4h at 37°C.

D) RP-HPLC chromatogram of [^{177}Lu]Lu-ePSMA-DM1 incubated in PBS for 24h at 37°C.

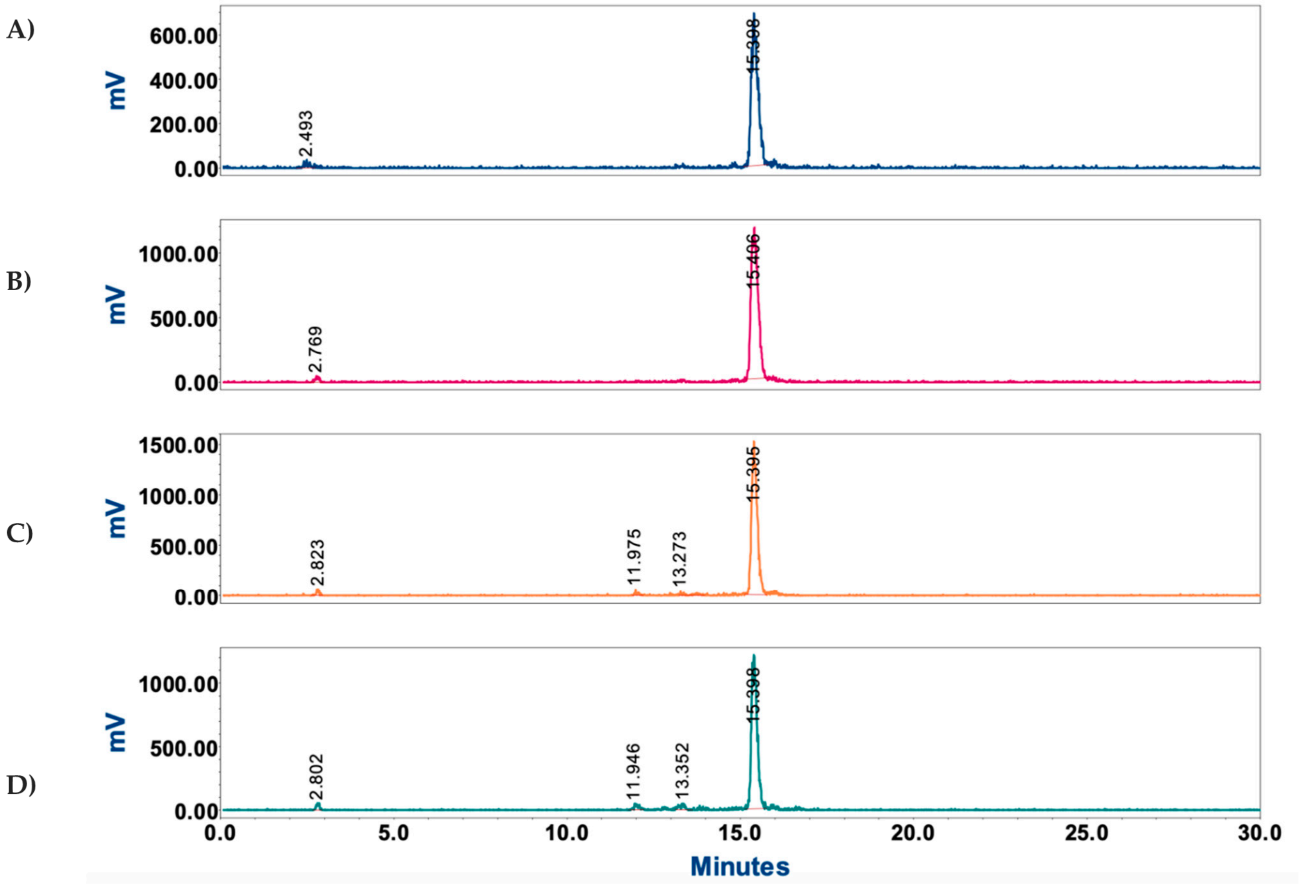

Figure S6. A) RP-HPLC chromatogram of [^{177}Lu]Lu-ePSMA-DM1. The peak at 15.308 minutes represents the compound [^{177}Lu]Lu-ePSMA-DM1. The peak at 2 minutes represents free ^{177}Lu.

B) RP-HPLC chromatogram of [^{177}Lu]Lu-ePSMA-DM1 incubated in mouse serum for 1h at 37°C.

C) RP-HPLC chromatogram of [^{177}Lu]Lu-ePSMA-DM1 incubated in mouse serum for 4h at 37°C.

D) RP-HPLC chromatogram of [^{177}Lu]Lu-ePSMA-DM1 incubated in mouse serum for 24h at 37°C.

NAALADase assay (binding affinity) of ePSMA-DM1 and the reference PSMA-617

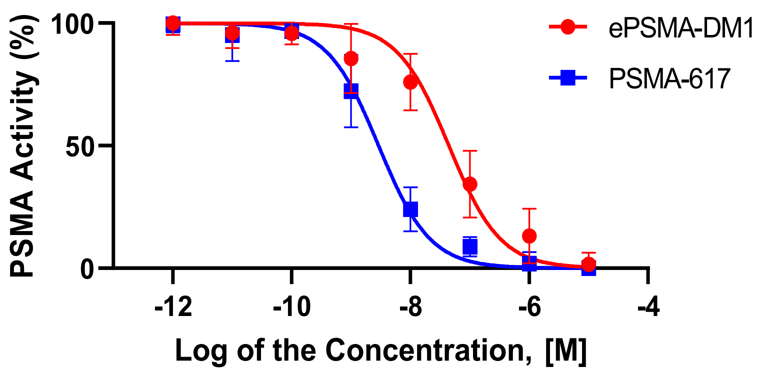

Figure S7. Binding affinity of ePSMA-DM1 towards PSMA, determined via an enzymatic NAALADase assay.

Ex vivo biodistribution of [^{111}In]In-ePSMA-DM1

Table 1. *Ex vivo* biodistribution of [^{111}In]In-ePSMA-DM1 (n=4). Organs were harvested and measured for their radioactivity in previously weighed tubes in order to determine the % ID/g. Activity measured was corrected for the time of injection and decay.

Organ	1h	4h	4h block	24h
Blood	1.43 ± 0.04	0.37 ± 0.01	0.30 ± 0.04	0.04 ± 0.00
Tumor	2.39 ± 0.29	1.19 ± 0.22	0.80 ± 0.31	0.50 ± 0.14
Heart	0.65 ± 0.10	0.23 ± 0.04	0.18 ± 0.00	0.10 ± 0.02
Lung	1.34 ± 0.18	0.40 ± 0.03	0.51 ± 0.26	0.25 ± 0.12
Liver	0.68 ± 0.00	0.63 ± 0.02	0.56 ± 0.05	0.39 ± 0.04
Spleen	1.32 ± 0.28	0.47 ± 0.08	0.32 ± 0.02	0.27 ± 0.01

Stomach	0.80 ± 0.61	0.47 ± 0.32	0.30 ± 0.24	0.05 ± 0.02
Intestines	0.56 ± 0.19	0.45 ± 0.20	0.56 ± 0.35	0.12 ± 0.03
Pancreas	0.43 ± 0.11	0.17 ± 0.01	0.14 ± 0.02	0.09 ± 0.01
Kidney	33.97 ± 2.54	31.55 ± 1.78	24.02 ± 1.46	13.23 ± 0.95
Muscle	0.30 ± 0.06	0.21 ± 0.16	0.10 ± 0.01	0.07 ± 0.01
Skin	1.37 ± 0.29	0.75 ± 0.12	0.57 ± 0.19	0.44 ± 0.05
Bone	0.73 ± 0.27	0.41 ± 0.06	0.33 ± 0.04	0.30 ± 0.03
Prostate	1.92 ± 0.87	0.46 ± 0.19	0.40 ± 0.18	0.16 ± 0.03
